# Conditioned Medium From the Stem Cells of Human Exfoliated Deciduous Teeth Ameliorates Neuropathic Pain in a Partial Sciatic Nerve Ligation Model

**DOI:** 10.3389/fphar.2022.745020

**Published:** 2022-03-31

**Authors:** Yao Liu, Fumiya Kano, Noboru Hashimoto, Linze Xia, Qiao Zhou, Xingmei Feng, Hideharu Hibi, Aya Miyazaki, Tsutomu Iwamoto, Yoshizo Matsuka, Zhijun Zhang, Eiji Tanaka, Akihito Yamamoto

**Affiliations:** ^1^ Department of Tissue Regeneration, Graduate School of Biomedical Sciences, Tokushima University, Tokushima, Japan; ^2^ Orthodontics and Dentofacial Orthopedics, Graduate School of Biomedical Sciences, Tokushima University, Tokushima, Japan; ^3^ Department of Stomatology, Affiliated Hospital, Nantong University, Nantong, China; ^4^ Department of Oral and Maxillofacial Surgery, Nagoya University Graduate School of Medicine, Nagoya, Japan; ^5^ Pediatric Dentistry, Graduate School of Biomedical Sciences, Tokushima University, Tokushima, Japan; ^6^ Department of Stomatognathic Function and Occlusal Reconstruction, Graduate School of Biomedical Sciences, Tokushima University, Tokushima, Japan; ^7^ Department of Human Anatomy, School of Medicine, Nantong University, Nantong, China

**Keywords:** neuropathic pain, dental pulp stem cells, macrophage, MCP-1, siglec-9, conditioned medium

## Abstract

In neuropathic pain (NP), injury or diseases of the somatosensory system often result in highly debilitating chronic pain. Currently, there is no effective drug for the complete and definitive treatment of NP. We investigated the therapeutic potential of conditioned medium (CM) derived from stem cells from human exfoliated deciduous teeth (SHED-CM) against NP using a mouse partial sciatic nerve ligation (PSL) model. Abnormal pain sensation, such as tactile allodynia and hyperalgesia, can be caused by PSL. In the behavioral test, intravenous administration of SHED-CM greatly improved the PSL-induced hypersensitivity. We found that treatment with SHED-CM resulted in the recruitment of M2 macrophages in the injured sciatic nerve and ipsilateral L4/L5 dorsal root ganglion and suppressed microglial activation in the spinal cord. Notably, specific depletion of the anti-inflammatory M2 macrophages by mannosylated-Clodrosome markedly reduced the antinociceptive effect of SHED-CM. Intravenous administration of CM from M2 induced by SHED-CM (M2-CM) ameliorated the PSL-induced hypersensitivity. We found that M2-CM directly suppressed the expression of nociceptive receptors as well as proinflammatory mediators in Schwann cells. Taken together, our data suggest that SHED-CM ameliorates NP through the induction of the analgesic anti-inflammatory M2 macrophages. Thus, SHED-CM may be a novel therapeutic candidate for NP.

## Introduction

Lesions or diseases of the somatosensory nervous system cause neuropathic pain (NP), characterized by allodynia and hyperalgesia. The development of efficacious treatments for NP is hindered by the diverse and complex pathophysiology of these diseases. There are no drugs to treat NP completely and definitively (Ji et al., 2014; Sommer et al., 2018).

After peripheral nerve injury, Schwann cells sense the axonal damage and recruit macrophages from blood vessels to the injured nerves. They produce several inflammatory mediators, which enhance the excitability of the dorsal root ganglion (DRG), and subsequently, astrocytes and microglia are activated in the CNS. Under these neuroinflammatory conditions, maladaptive plasticity alters the nociceptive signal processing, which is associated with the induction and maintenance of NP; hence, interventions targeting neuroinflammation are promising therapeutic candidates for NP (Argoff, 2011; Calvo et al., 2012; Ji et al., 2016).

The diverse activation states of macrophage-monocyte lineages play crucial roles in tissue homeostasis (Murray et al., 2014; Inoue and Tsuda, 2018). Macrophages are classified into M1 and M2 phenotypes: M1 phenotypes produce typical proinflammatory cytokines, chemokines, reactive oxygen species, and nitric oxide, which initiate inflammation and induce tissue destruction, whereas M2 phenotypes suppress inflammation by secreting anti-inflammatory cytokines and scavenging cellular debris (Gordon and Martinez, 2010). It has been shown that M2 phenotypes are therapeutic against NP (Kiguchi et al., 2015; Celik et al., 2020); however, the detailed mechanisms underlying their anti-NP action remain elusive.

Recently, mesenchymal stem cell therapy has shown great potential for NP in clinical and preclinical studies (Vickers et al., 2014; Asgharzade et al., 2020; Wang et al., 2020). Notably, in most of these studies, neurological function was recovered primarily through paracrine/trophic mechanisms. Stem cells secrete a broad range of trophic and immunomodulatory factors that can be collected as serum-free conditioned medium (CM) (Baraniak and McDevitt, 2010). The analgesic activity of CM prepared by adipose tissue-derived stem cells or bone marrow-derived stem cells have been reported (Brini et al., 2017; Gama et al., 2018; De Gregorio et al., 2020). We reported that intravenous administration of CM derived from stem cells from human exfoliated deciduous teeth (SHED-CM) exerts remarkable therapeutic effects on CNS and PNS injuries, neurodegenerative and autoimmune diseases (Yamagata et al., 2013; Matsubara et al., 2015; Mita et al., 2015; Shimojima et al., 2016). The powerful antinociceptive effect of dental pulp stem cells (DPSCs) in diabetes-induced neuropathy (Guimaraes et al., 2013; Makino et al., 2019; Xie et al., 2019) and osteoarthritis-induced pain (Ogasawara et al., 2020) has also been reported. However, the detailed mechanisms by which DPSCs or SHED-CM ameliorate the pain remain unclear. Furthermore, the therapeutic effect of SHED-CM in nerve injury-induced NP has not been investigated.

In our previous study, we have identified a set of M2 inducers, monocyte chemoattractant protein-1 (MCP-1) and the secreted ectodomain of sialic acid-binding Ig-like lectin-9 (sSiglec-9), by secretome analysis of SHED-CM (Matsubara et al., 2015). MCP-1 is a chemokine that recruits immune cells to inflamed tissues (Bachmann et al., 2006), and Siglecs are a large family of sialic-acid-binding type-I transmembrane immunoglobulin-like lectins that modulate immune signaling in various types of immune cells (Crocker et al., 2007). We have reported that MCP-1/sSiglec-9 treatment promoted substantial recovery of the motor function after spinal cord injury in rats (Matsubara et al., 2015) and facial nerve transection (Kano et al., 2017), from acute liver failure (Ito et al., 2017) and bone regeneration in rat calvarial bone defects (Ishikawa et al., 2019) however, their anti-NP activity has not been examined.

Herein, we examined the therapeutic potential of intravenously administered SHED-CM against NP induced by partial sciatic nerve ligation (PSL) and its analgesic mechanisms.

## Materials and Methods

### Animals

All animal experiments were approved by the Animal Research Committees of Tokushima University (Permit No: T2020-09) and Nantong University (Permit No: S20210308-004). All animal experiments were conformed to the ethical guidelines of the International Association for the Study of Pain (Zimmermann, 1983) and ARRIVE. Male ICR mice (Charles River, Yokohama, Japan) aged 7–11 weeks were used in all the experiments. All the mice were housed in plastic cages under standard laboratory conditions (12 h dark/light cycle, temperature controlled between 23 and 24°C) and provided with water and food *ad libitum*. An overview of the experimental design and workflow is presented in Supplementary Figure S1.

### Partial Sciatic Nerve Ligation and Drug Administration

To induce NP, PSL was performed according to a well-characterized method (Seltzer et al., 1990). Briefly, mice were deeply anesthetized with a mixture of 5.0 mg/ml Vetorphale (Meiji Seika, Tokyo, Japan), 1.0 mg/ml Domitor (Zenoag, Fukushima, Japan), 5.0 mg/ml midazolam (Sandoz, Yamagata, Japan) and diluted with distilled water for injection (79% of the total volume). All the mice were anesthetized with an intraperitoneal injection of the three mixtures (10 ml/kg). The right sciatic nerve (SCN) was exposed at the high thigh level. The dorsum of the SCN was carefully freed from the surrounding tissues, and its dorsal aspect was carefully supported with a glass rod without pressing the nerve against the underlying structures. A 3/8 curved mini-needle with 5–0 silk suture was inserted into the nerve, and 1/3–1/2 of the nerve thickness was tightly ligated. The incision was then closed using three to four skin sutures (4–0). In the sham controls, the SCN was exposed with a small incision but was not ligated before the incision was closed.

**FIGURE 1:**
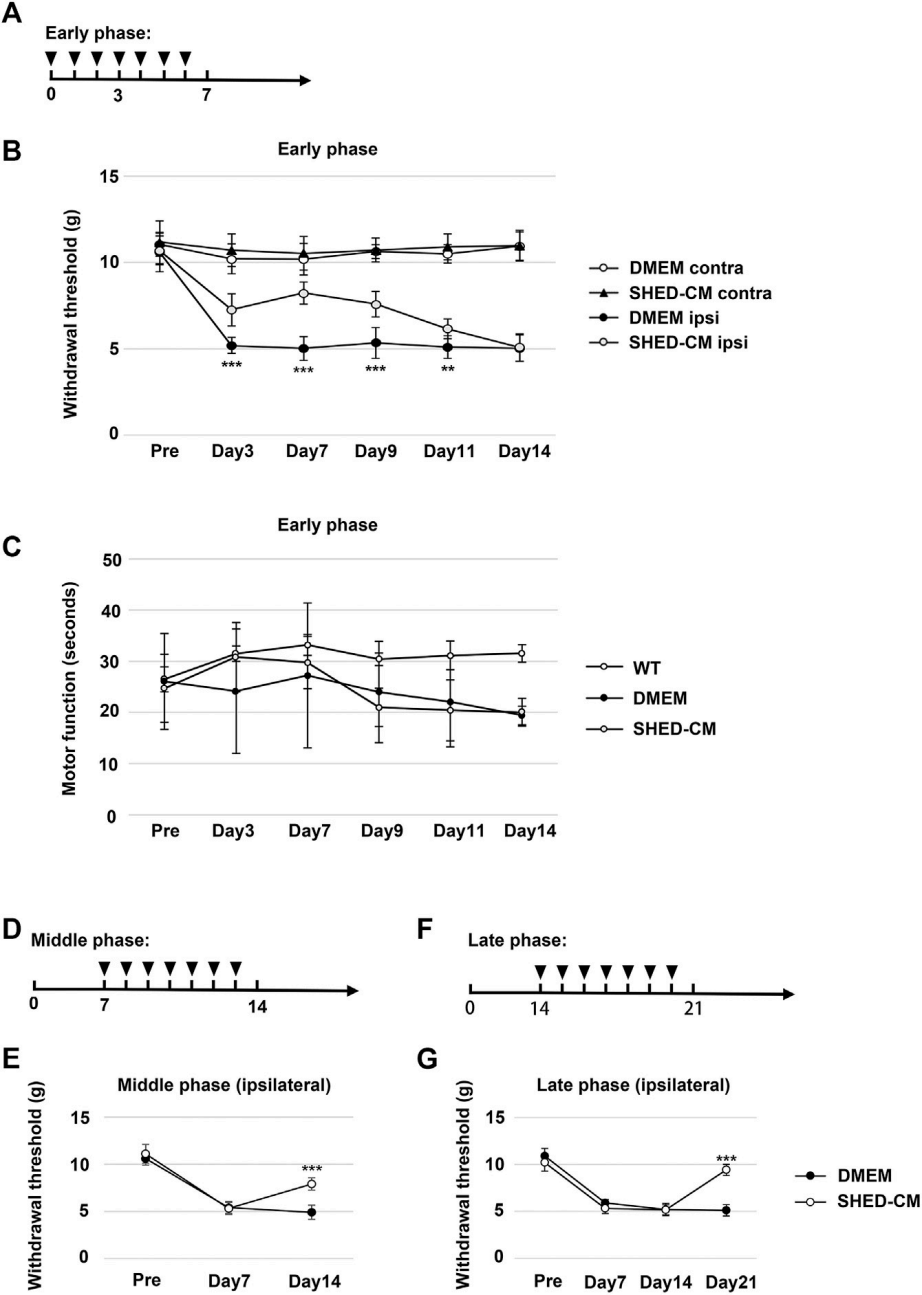
Intravenous administration of SHED-CM attenuates partial sciatic nerve ligation (PSL)-induced neuropathic pain (NP). **(A)** Time course of SHED-CM treatment in the early phase model. **(B)** The ipsilateral paw withdrawal thresholds in the early phase. Student’s *t*-test (*n* = 9 in each group). **(C)** Rotarod test in the early phase model (*n* = 10 in each group). **(D)** Time course of SHED-CM treatment in the middle phase model. **(E)** The ipsilateral paw withdrawal thresholds in the middle phase. Student’s *t*-test (*n* = 5 in each group). **(F)** Time course of SHED-CM treatment in the late phase model. **(G)** The ipsilateral paw withdrawal thresholds in the late phase. Student’s *t*-test (*n* = 5 in each group). Data represent the mean ± SD. **p < 0.01, ***p < 0.001 for the SHED-CM vs. DMEM.

Recombinant MCP-1 (Peprotech, London, United Kingdom) and sSiglec-9 (R&D Systems, Minneapolis, MN, United States) were diluted with phosphate-buffered saline (PBS) to a concentration of 500 ng/ml for each protein. CM or MCP-1/sSiglec-9 was intravenously administered from tail vein, during day 0 to day 6 in the early phase model, day 7 to day 13 in the middle phase model, and day 14 to 20 in the late phase model (Figures 1A,D,F).

### Stem Cells From Human Exfoliated Deciduous Teeth-Conditioned Medium Preparation

SHEDs were isolated and cultured as described previously (Sakai et al., 2012). In brief, deciduous teeth from 6- to 12-year-old individuals were collected at the Nagoya University and Tokushima University Hospital. We not only confirmed donor’s intentions, but also obtained the consent of their parents or guardians. This study was approved by the Institutional Ethical Committee of Nagoya University and Tokushima University Hospital and performed in accordance with the principles of the Declaration of Helsinki (Permit No H-73 and No: 3268 for Nagoya and Tokushima University, respectively). All participants provided written informed consent. After separating the crown and root, the dental pulp was isolated and digested in a solution of 3 mg/ml collagenase type I and 4 mg/ml dispase for 1 h at 37°C. Single-cell suspensions (3 × 10^4^ cells/ml) were plated on culture dishes in Dulbecco’s modified Eagle’s medium (DMEM, Sigma, St. Louis, MO, United States) supplemented with 10% fetal calf serum (Sigma), and then incubated at 37°C in an atmosphere of 5% CO_2_ at 100% relative humidity. The human skin fibroblast line, derived from a 36-year-old individual, was obtained at passage 12 from the Health Science Research Resources Bank (Osaka, Japan). The seeding density of SHED (passage 9) and fibroblasts (passage 13) for CM preparation was 1 × 10^5^.

After several passages, SHEDs or fibroblasts at 70–80% confluency were washed twice with PBS, and the culture medium was replaced with serum-free DMEM. After a 48 h incubation, the medium was collected and centrifuged for 3 min at 440 × *g*. The supernatant was then collected and centrifuged for 3 min at 17,400 × *g*. The resulting supernatant was used as SHED-CM or fibroblast-CM (Fibro-CM) in different experiments. The protein concentration in the SHED-CM and Fibro-CM was measured using the bicinchoninic acid protein assay kit (Pierce, Rockford, IL, United States) and adjusted to 3 μg/ml with DMEM.

### Behavioral Testing

All the candidate mice were habituated to the testing environment 2 h daily for at least 2 days before baseline testing. Mice with stable data were chosen for the subsequent von Frey test. They were individually placed on a 5 × 5 mm metal mesh floor covered with small plastic containers and allowed approximately 2–3 h for acclimatizing to the environment before testing.

PSL-induced tactile allodynia was evaluated by the von Frey test using the up-down method according to a method described in a previous study (Chaplan et al., 1994). The investigator was blinded to the treatment groups. Mechanical hypersensitivity was measured using an electronic von Frey device (Ugo Basile S.R.L, Varese, Italy). The von Frey filament was applied to the middle of the plantar surface of the hind paw, and withdrawal responses were measured five times for each hind paw. A positive reaction was recorded if the mice exhibited a brisk paw withdrawal reaction upon stimulation. Motor function was tested using a rotarod (LE8500, Bio Research Center, Nagoya, Japan). Before the rotarod test, the mice were habituated with the apparatus. The rotating cylinder was accelerated at 4 to 40 rotations per minute and observed for 1 min. The time until the mice fell from the rod was recorded.

### Real-Time Quantitative Polymerase Chain Reaction

Fresh sciatic nerve, 1 cm length covering the ligation site on early phase day 3, or human Schwann cells were harvested and placed in a 1.5 ml RNase-free tube and homogenized with Isogen (Nippon Gene, Tokyo, Japan). Chloroform was added, followed by centrifugation at 4°C for 30 min. The aqueous phase containing RNA was transferred to a fresh tube, and RNA was precipitated with isopropyl alcohol and 75% ethanol by centrifugation. Purified total RNA was quantified using a spectrophotometer (Denovix, Wilmington, DE, United States), and RNA integrity was checked on 1.5% agarose gels. The purified total RNA was converted to cDNA by reverse transcription using M-MLV reverse transcriptase (Thermo Fisher, Carlsbad, CA, United States) and random primers (Thermo Fisher). cDNA was used as a template for qPCR with the THUNDERBIRD SYBR qPCR Mix (Toyobo, Osaka, Japan), and the amplification was performed on the StepOnePlus Real-Time PCR System (Applied Biosystems, Foster City, CA, United States). The primers used are listed in Table 1. The fluorescence intensity of the intercalated SYBR Green was measured and normalized to that of glyceraldehyde-3-phosphate dehydrogenase (GAPDH).

**TABLE 1:** Primer sequences used in the qRT-PCR.

Gene		Primer Sequence
mouse GAPDH	Forward	AAC​TTT​GGC​ATT​GTG​GAA​GG
mouse GAPDH	Reverse	GGA​TGC​AGG​GAT​GAT​GTT​CT
mouse F4/80	Forward	CCA​GAA​GGC​TCC​CAA​GGA​T
mouse F4/80	Reverse	TCT​GCT​CAC​TTT​GGA​GTA​TCA​AGT​C
mouse TNF-α	Forward	CCC​TTT​ACT​CTG​ACC​CCT​TTA​TTG​T
mouse TNF-α	Reverse	TGT​CCC​AGC​ATC​TTG​TGT​TTC​T
mouse IL-1β	Forward	CCT​CTG​ATG​GGC​AAC​CAC​TT
mouse IL-1β	Reverse	TGC​TGC​CTA​ATG​TCC​CCT​TG
mouse iNOS	Forward	AGC​CAA​GCC​CTC​ACC​TAC​TTC
mouse iNOS	Reverse	GCC​TCC​AAT​CTC​TGC​CTA​TCC
mouse CD206	Forward	CAG​GTG​TGG​GCT​CAG​GTA​GT
mouse CD206	Reverse	TGT​GGT​GAG​CTG​AAA​GGT​GA
mouse Arginase-1	Forward	CTC​CAA​GCC​AAA​GTC​CTT​AGA​G
mouse Arginase-1	Reverse	GGA​GCT​GTC​ATT​AGG​GAC​ATC​A
mouse Ym-1	Forward	CTC​TCC​AGA​AGC​AAT​CCT​GAA​GAC
mouse Ym-1	Reverse	GCC​CAA​CTG​GTA​TAG​TAG​CAC​ATC
mouse BDNF	Forward	TTA​ATC​GGC​TTC​ACA​GGA​GAC​A
mouse BDNF	Reverse	AGA​ACG​AAC​AGA​AAC​GAA​GAA​GAG​A
mouse NGF	Forward	ACA​GAC​ATC​AAG​GGC​AAG​GAG
mouse NGF	Reverse	GCA​CCC​ACT​CTC​AAC​AGG​ATT
mouse GDNF	Forward	TGC​TAA​AGG​AAA​GGG​TCA​GGA​G
mouse GDNF	Reverse	GCT​CAG​ATG​GAT​AGA​AGA​GGA​GAT​G
mouse TGF-β1	Forward	CCA​CCT​GCA​AGA​CCA​TCG​AC
mouse TGF-β1	Reverse	CTG​GCG​AGC​CTT​AGT​TTG​GAC
mouse IL-10	Forward	GCT​CTT​ACT​GAC​TGG​CAT​GAG
mouse IL-10	Reverse	CGC​AGC​TCT​AGG​AGC​ATG​TG
human GAPDH	Forward	CTG​GGC​TAC​ACT​GAG​CAC​C
human GAPDH	Reverse	AAG​TGG​TCG​TTG​AGG​GCA​ATG
human TRPA1	Forward	AAGCCGTTGCGCTTCTTC
human TRPA1	Reverse	GAC​ATT​CAT​CCC​ATC​TTT​TGC​TC
human TNF-α	Forward	GAG​GCC​AAG​CCC​TGG​TAT​G
human TNF-α	Reverse	CGG​GCC​GAT​TGA​TCT​CAG​C
human IL-1β	Forward	CCT​GTC​CTG​CGT​GTT​GAA​AG
human IL-1β	Reverse	GGG​AAC​TGG​GCA​GAC​TCA​AA
human MCP-1	Forward	AAT​CAA​TGC​CCC​AGT​CAC​CT
human MCP-1	Reverse	CCT​GAA​CCC​ACT​TCT​GCT​TG

### Immunohistochemistry

Mice from each group were deeply anesthetized before intracardiac perfusion with PBS, followed by 4% paraformaldehyde. After that, fixed SCN, L4/L5 DRG, and L3-L4 spinal cord were collected from mice, post-fixed in 4% paraformaldehyde, and dehydrated overnight in 25% sucrose at 4°C. Frozen tissue embedded in the OCT compound (Sakura Finetek, Torrance, CA, United States) and SCN and DRG were cut longitudinally into 10 μm thick sections, and the spinal cord was cut into 30 μm thick sections and mounted on glass slides. The sections were permeabilized with 0.3% Triton X-100 in PBS for 15 min, blocked with 5% normal donkey serum at 25–27°C for 1 h and incubated overnight with the following primary antibodies: rat anti-F4/80 (1:800, ab6640, Abcam, Cambridge, United Kingdom), rabbit anti-CD206 (1:1000, ab64693, Abcam), rabbit anti-Iba1 (1:1000, Wako, Osaka, Japan), rabbit anti-GFAP (1:1000, ab7260, Abcam), mouse anti-S100β (1:500, AMAB91038, Sigma), and rabbit anti-TNF-α (1:1000, ab9739, Abcam). The sections were washed with 0.3% Triton X-100 in PBS on the following day and incubated with fluorescence-conjugated secondary antibodies (anti-rabbit Alexa Fluor 488, Thermo Fisher, Eugene, OR, United States; anti-rabbit Alexa Fluor Plus 555, Thermo Fisher; anti-rat Alexa Fluor 555, ab150166, Abcam; anti-mouse Alexa Fluor Plus 488, Thermo Fisher) at ∼25°C for 1 h. The sections were washed with 0.3% Triton X-100 in PBS and incubated with DAPI (1:500, Sigma) at room temperature for 5 min. Finally, the sections were mounted on a slide using a mounting medium and covered with a cover slip. The images of the tissues were captured using a universal fluorescence microscope (BZX700, Keyence, Osaka, Japan) and a confocal microscope (FV1200, Olympus, Tokyo, Japan). CD206/F4/80- and TNF-α/S100β-positive cells 2 mm distal to the injury site in proximal SCN and CD206/F4/80- positive cells in DRG were counted at ×400 magnification. Iba1-positive cells in spinal cord dorsal horn and ventral horn were counted at ×200 magnification. All the positive cells were counted from at least three non-overlapping sections obtained from 8 animals per group.

### M2 Macrophage Depletion *In Vivo*

The mannosylated-Clodrosome Macrophage Depletion Kit (m-Clodrosome: 5 mg/ml Clodronate Disodium Salt, 18.8 mg/ml L-α-Phosphatidylcholine, 4.2 mg/ml Cholesterol, and 1 mg/ml 4-Aminophenyl-alpha-D-Mannopyranoside; Encapsula NanoSciences, Brentwood, TN, United States) was used to deplete M2 macrophages from the PSL mice. After PSL, SHED-CM was injected daily for 7 consecutive days, and from day 4 to day 6, 2 mg/kg mannosylated-Clodrosome (m-Clo) was orally administered. Control mice received mannosylated liposomes without Clodronate Disodium Salt (m-Encapsome: 18.8 mg/ml L-α-Phosphatidylcholine, 4.2 mg/ml Cholesterol, and 1 mg/ml 4-Aminophenyl-alpha-D-Mannopyranoside) in the same volume. m-Clodrosome depletes M2 macrophages, which express a mannose receptor (MR, MMR, or CD206). Ten animals per group were sacrificed for tissue collection 7 days afte PSL.

### M2-CM Preparation

Bone marrow cells from the femurs of 8-weeks-old male mice were plated on 6 cm dishes (2.0 × 10^6^ cells per dish) and differentiated into macrophages in DMEM supplemented with 20 ng/ml macrophage colony-stimulating factor (MCSF, R&D) at 37°C in an atmosphere of 5% CO_2_ for 7 days. The macrophages were then incubated with serum-free DMEM and SHED-CM for 24 h. Phase contrast images of the induced macrophages were captured using a digital microscope camera (Leica DFC290 HD, Leica, Wetzlar, Germany). The mRNA expression of M2-type cell markers or trophic factors was examined by qPCR analysis (Supplementary Figure S2). SHED-CM-induced macrophages were designated as M2 macrophages. The induced macrophages were washed twice with PBS, and the culture medium was replaced with serum-free DMEM. After 24 h of incubation, the medium was collected and centrifuged for 5 min at 440 × *g*. The supernatant was then collected and centrifuged for 5 min at 17,400 × *g*. The resulting supernatant was used as M2-CM in subsequent *in vivo* and *in vitro* experiments.

### Activation of Human Schwann Cell *In Vitro*

Human Schwann cells (Passage 5) were obtained from ScienCell Research Laboratories (Cat. #1700; Carlsbad, CA, United States). The cells were seeded in 6 cm dishes at a density of 3 × 10^5^ cells per dish. After incubation for 24 h in 3 ml serum-free DMEM or M2-CM with 10 ng/ml TNF-α, the cells were harvested for gene analysis.

### Flow Cytometry

SHED-CM-treated mouse bone marrow-derived macrophages were scraped with a cell scraper (IWAKI, Tokyo, Japan) and centrifuged at 400 × *g* for 5 min. The cells were washed with the FACS incubation buffer (25 μg/ml DNase I (Sigma) and 1% Bovine serum albumin (BSA; Wako) containing PBS and incubated with the CD206-PE and F4/80-FITC antibodies (Biolegend, San Diego, CA, United States) in the FACS incubation buffer. Subsequently, they were analyzed using a flow cytometer (FACS Canto, BD Biosciences, San Jose, CA, United States).

### Statistical Analyses

The Student’s *t*-test was used for statistical analysis of the mean values of the two groups, and Tukey’s multiple comparison test was used to test the difference between the means of three or more groups. All statistical analyses were performed using the statistical analysis software R (version 4.0.2; available as a free download from https://www.r-project.org/). A *p*-value of <0.05 was used to declare the statistical significance.

## Results

### Stem Cells From Human Exfoliated Deciduous Teeth-Conditioned Medium Prevents NP and Maintains Locomotor Function After PSL

We tightly ligated 1/3 to 1/2 of the SCN on the right side of mice to induce NP. A decrease in the threshold for tactile stimuli was observed after nerve ligation. The threshold was reached at least 3 days after PSL and was maintained at the level for at least 2 weeks. Daily intravenous administration of SHED-CM, but not Fibro-CM, immediately after PSL (early phase), inhibited PSL-induced mechanical allodynia (Figures 1A,B, Supplementary Figure S3A). These antinociceptive effects of SHED-CM were detected even 3 days after the administration of SHED-CM. In the von Frey test, the threshold for the right hindpaw on day 3 was 6.73 ± 1.50 g in the SHED-CM group and 4.29 ± 1.49 g in the DMEM group. On day 7, the threshold of the SHED-CM group was 8.39 ± 0.99 g, which was significantly higher than that in the DMEM group (4.30 ± 1.78 g). Furthermore, the antinociceptive effects of SHED-CM disappeared after 7 days from the last injection. Locomotor function analyzed by Rotarod testing revealed reduced motor function in DMEM-treated PSL mice, whereas that of SHED-CM mice was equivalent to uninjured wild type mice during the treatment period (days 1–7), which was immediately decreased after the last SHED-CM injection (Figure 1C).

In previous study, it has been reported that the development of NP occurred within a week after PSL surgery when the number of Iba1^+^ activated microglia increased in spinal cord. In the subsequent maintenance phase, the number of microglia was decreased; however, that of GFAP^+^ activated astrocytes greatly increased (Tanga et al., 2004). In our experimental setting, the early phase and middle/late phase were development and maintenance phase, respectively (Supplementary Figure S4). Furthermore, to evaluate the antinociceptive activity of SHED-CM against the maintenance of PSL-induced NP, we carried out the von Frey test in middle and late phases. SHED-CM treatment exhibited significant antinociceptive effects in both the middle and the late phase model. In the middle phase, the thresholds of the SHED-CM and DMEM groups on 14 days after PSL were 8.07 ± 0.84 g and 4.82 ± 0.92 g, respectively, (Figures 1D,E). In the late phase, the thresholds on 21 days after PSL were 9.47 ± 0.74 and 5.07 ± 0.67 g, respectively (Figures 1F,G). However, during the SHED-CM treatment, the von Frey test data of the contralateral side did not show any change (Supplementary Figure S3B). These results demonstrated the potential of SHED-CM to inhibit both the development and maintenance of NP.

### Stem Cells From Human Exfoliated Deciduous Teeth-Conditioned Medium Treatment Induces M2-Polarized Macrophages in Ipsilateral DRG

Immunohistochemical analysis of the ipsilateral L4/L5 DRG, 7 days after PSL, revealed that SHED-CM treatment significantly increased the number of CD206^+^ F4/80^+^ M2 macrophages compared with the DMEM control treatment (Figure 3E). The cell count analysis showed that the number of M2 cells in the SHED-CM group was significantly higher than that in the DMEM group (Figure 3F), indicating that M2 not only accumulated in the ipsilateral SCN but also in the ipsilateral DRG.

### Stem Cells From Human Exfoliated Deciduous Teeth-Conditioned Medium Treatment Induces M2-Polarized Macrophages After PSL

**FIGURE 2:**
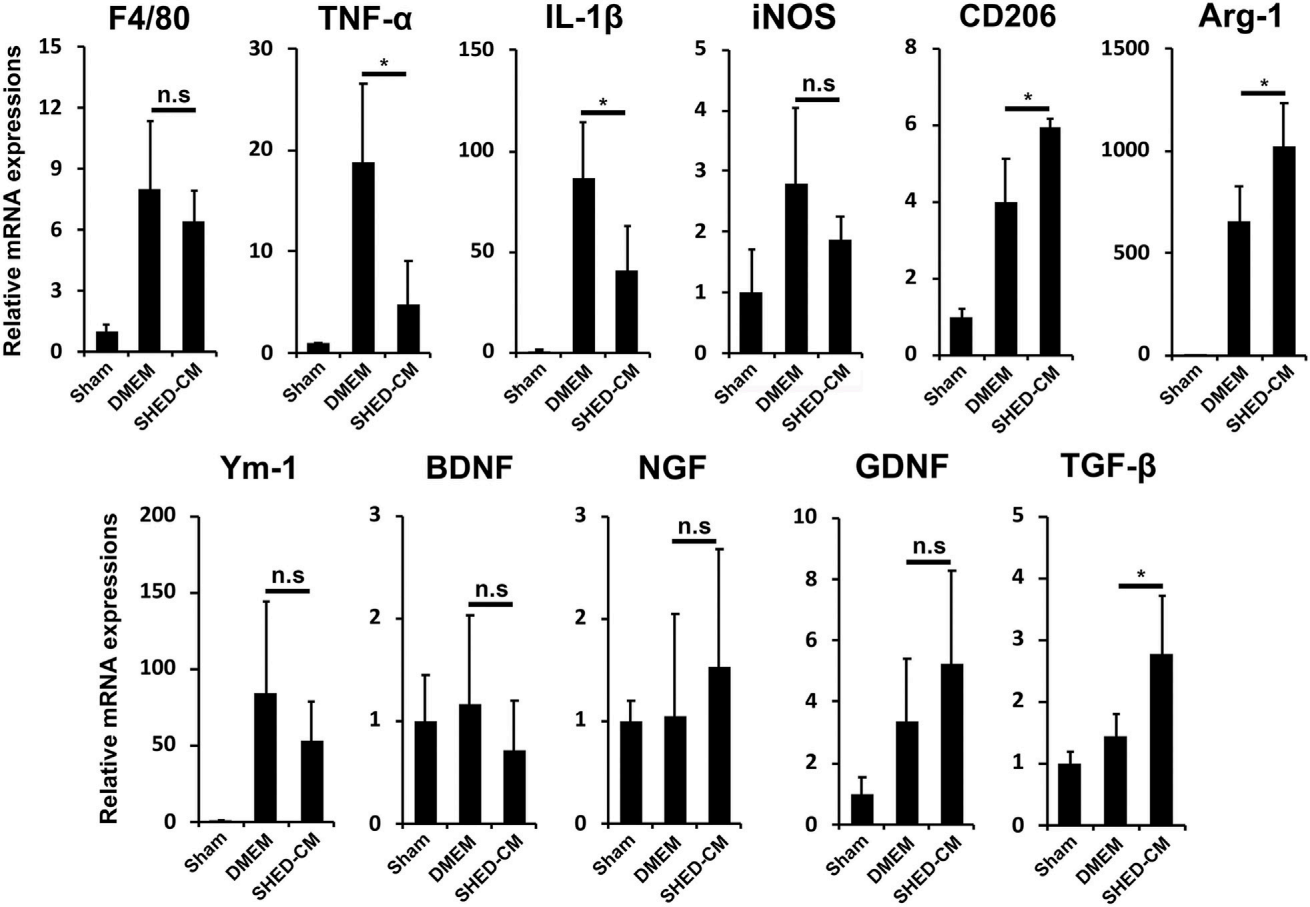
SHED-CM treatment converts proinflammatory microenvironment to an anti-inflammatory and tissue-protective microenvironment. qPCR analysis of the indicated mRNAs in the sciatic nerves 3 days after PSL in the early phase model. Student’s *t*-test (*n* = 4). Data represent the mean ± SD. *p < 0.05.

To investigate the analgesic mechanism of SHED-CM, we examined the mRNA expression profiles of genes involved in pro- and anti-inflammatory responses in the early phase PSL. Three days after PSL, expressions of inflammatory genes, TNF-α, IL-1β, and inducible nitric oxide synthase (iNOS), was increased, which was suppressed by SHED-CM treatment. The SHED-CM treatment had no effect on the expression of pan macrophage markers F4/80, but increased significantly M2-specific molecules, CD206 and arginase-1 (Arg-1). The SHED-CM treatment also elevated the expression of an array of neurotrophic and immunosuppressive factors, nerve growth factor (NGF), glial cell-derived neurotrophic factor (GDNF), and transforming growth factor-β1 (TGF-β1) (Figure 2). These results showed that the SHED-CM treatment converted the proinflammatory microenvironment of PSL to an anti-inflammatory and tissue-protective microenvironment.

**FIGURE 3:**
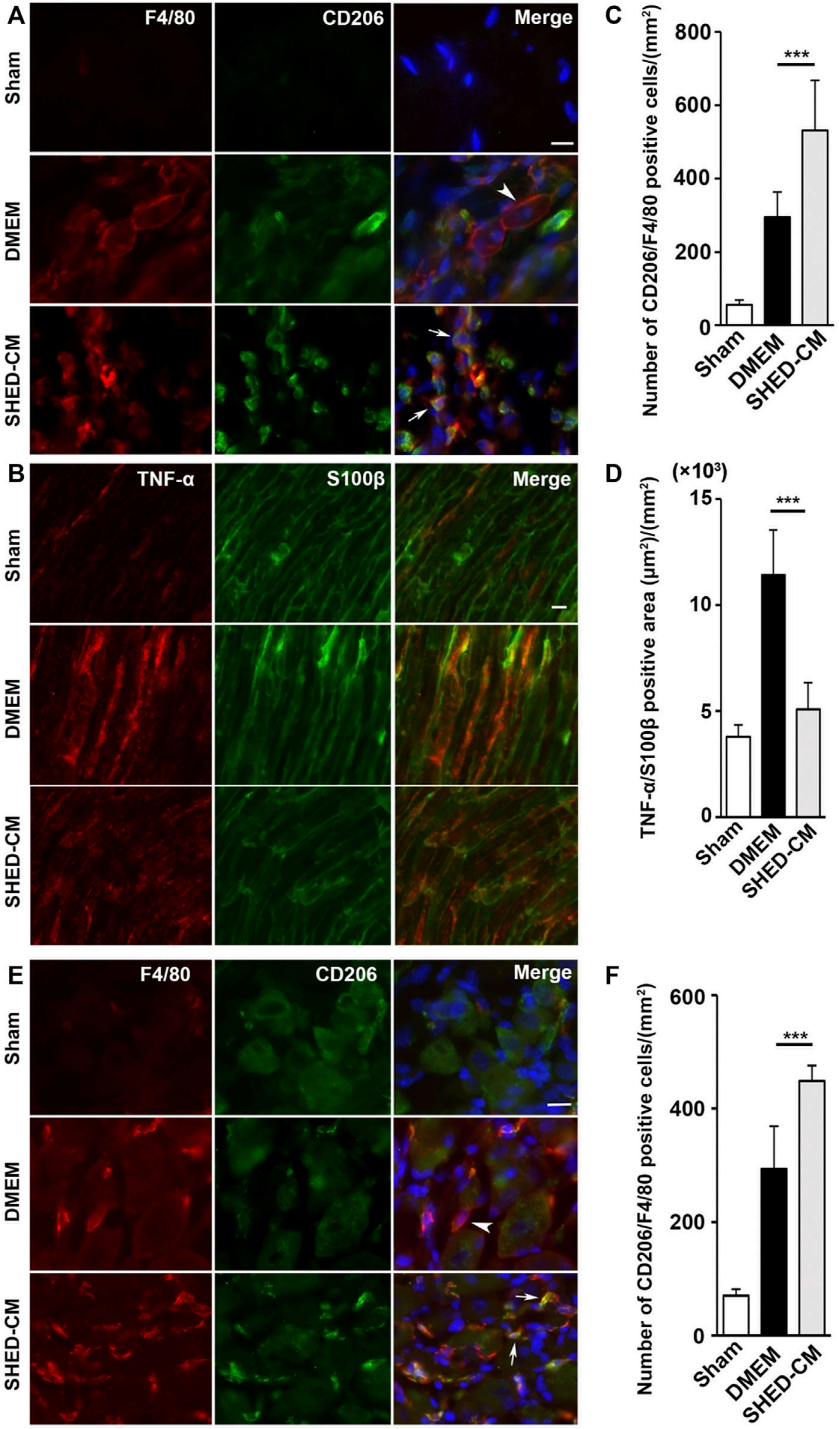
SHED-CM treatment induces M2 macrophages. **(A,B)** Representative images of immunofluorescent staining of CD206, F4/80 **(A)** and TNF-α, S100β **(B)** in SCN. **(C,D)** Quantification of CD206^+^ F4/80^+^ cell numbers **(C)** and TNF-α^+^ S100β^+^ area **(D)** in SCN. **(E)** Images of immunofluorescent staining of CD206, F4/80 in L4/5 DRG. **(F)** Quantification of CD206^+^ F4/80^+^ cell numbers in L4/5 DRG. Scale bar: 10 μm in **(A,B,** **E)**. At least 3 non-overlapping sections from 8 animals per group were used to determine the positive cells. Data represent the mean ± SD. Student’s *t*-test (*n* = 8, per group); ***p < 0.001. Arrowheads and arrows in **(A)** and **(E)** indicate CD206^-^F4/80^+^ and CD206^+^ F4/80^+^, respectively.

Furthermore, immunohistochemical analysis revealed that the SHED-CM treatment significantly increased the number of CD206^+^ F4/80^+^ macrophages in the proximal side of the ligated SCN. However, TNF-α^+^S100β^+^ proinflammatory Schwann cells and TNF-α^+^F4/80^+^ M1 macrophages were greatly decreased by the SHED-CM treatment (Figures 3A–D; Supplementary Figures S5, S6).

### Stem Cells From Human Exfoliated Deciduous Teeth-Conditioned Medium Attenuates PSL-Induced Microglial Activation in the Spinal Cord

**FIGURE 4:**
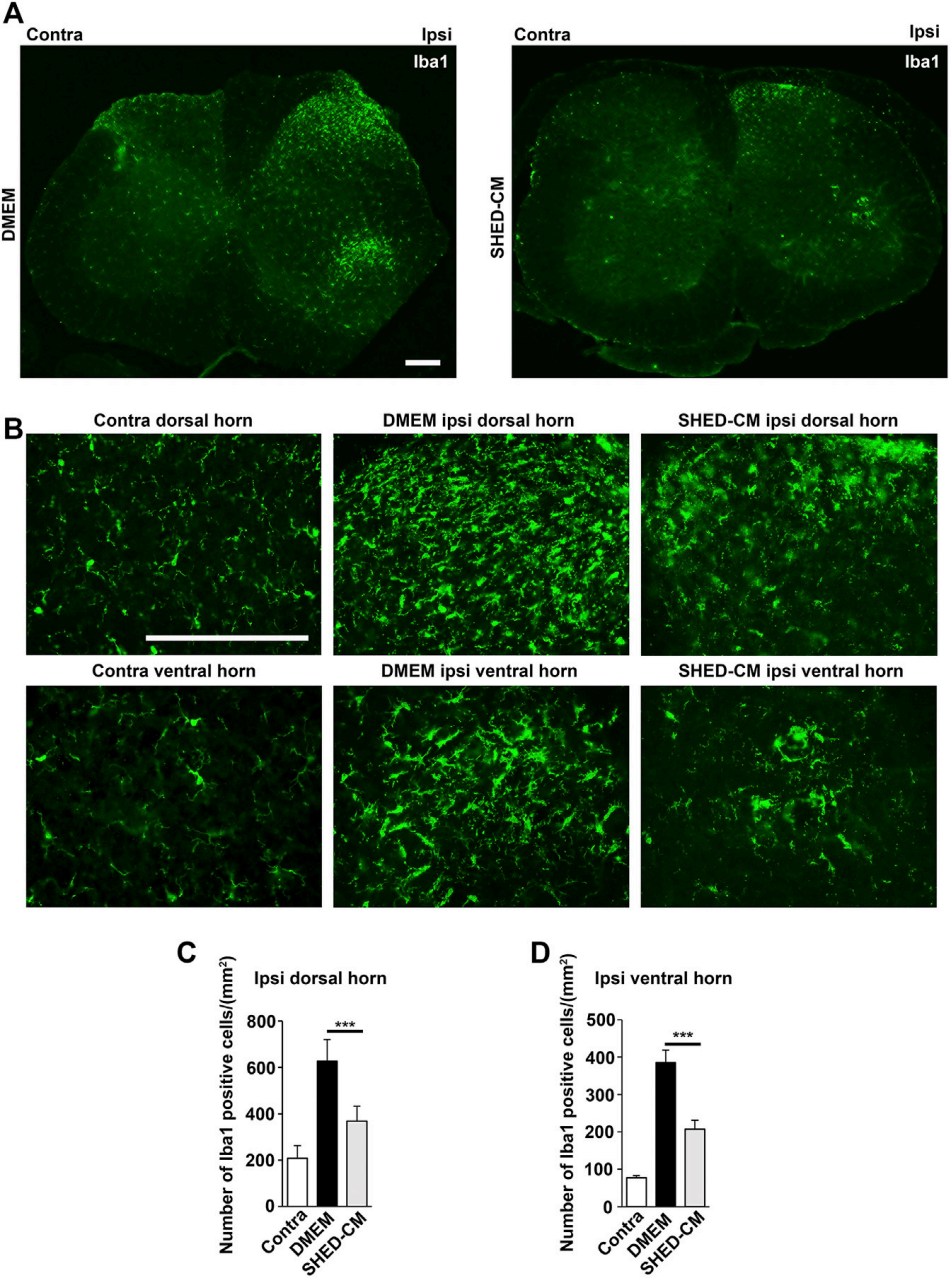
SHED-CM treatment suppresses the activation of microglia. **(A,B)** Representative images of immunofluorescent staining of Iba1 in the contralateral and ipsilateral horn of L3/4 SP 7 days after PSL in the early phase model. **(C,D)** Iba1^+^ cell numbers in the **(C)** dorsal and **(D)** ventral horn of L3/4 SP. Scale bar: 200 μm in **(A,B)**. Student’s *t*-test (*n* = 7–10, per group). Data represent the mean ± SD. ***p < 0.001.

Next, we examined microglial activation 7 days after PSL. As shown in Figure 4, the numbers of Iba1^+^ microglia in the L3/4 ipsilateral dorsal and ventral horn were increased compared with that in the contralateral side. They showed activated microglial morphology with a hypertrophied soma and thicker and retracted processes in the ipsilateral side, whereas that of the contralateral side seemed to be at the quiescent stage with smaller soma and ramified processes. Notably, in the SHED-CM-treated group, the number of Iba1^+^ microglia in both ipsilateral dorsal and ventral horn was reduced, and their morphologies were very similar to those on the contralateral side (Figures 4A–D). These results demonstrated that SHED-CM treatment suppressed the PSL-induced microglial activation in the spinal cord.

### M2 Macrophages Induced by SHED-CM are Required for Its Antinociceptive Activity

**FIGURE 5:**
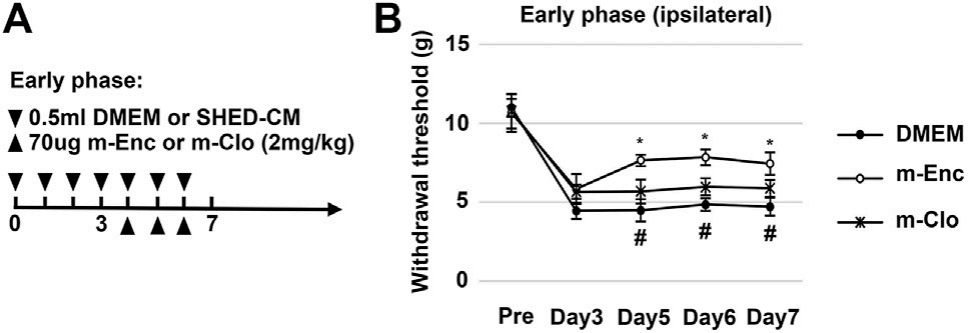
Effects of M2 depletion on SHED-CM-mediated behavioral recovery after PSL. **(A)** Time course of M2 depletion in the early phase model. After PSL, mice were daily administered with either 0.5 ml of SHED-CM or DMEM. From day 4 to 6, mannosylated-Clodrosome (m-Clo; for M2 depletion) or mannosylated-Encapsome (m-Enc; negative control) were injected together with SHED-CM. **(B)** The ipsilateral paw withdrawal thresholds. m-Clo, but not m-Enc, significantly inhibits the antinociceptive effects of SHED-CM. Notably, the paw withdrawal threshold in the m-Clo group was significantly higher than that in the DMEM group. Tukey’s multiple comparisons of means (*n* = 10, per group). Data represent the mean ± SD. *p < 0.05, for the m-Clo vs. m-Enc. #*p* < 0.05 for the m-Clo vs. DMEM.

Next, we investigated the roles of M2 macrophages on the antinociceptive activity of SHED-CM by depleting them with m-Clo. The time course of M2 depletion in the early phase model is shown in Figure 5A. The results demonstrated that, on day 5, the antinociceptive effects of m-Clo group, but not m-Enc, was significantly decreased. On day 7, the von Frey test thresholds of the m-Clo, m-Enc, and DMEM groups were 5.49 ± 0.92, 7.90 ± 1.33, and 4.57 ± 1.00 g, respectively (Figure 5B). The thresholds of contralateral side of PSL treated with m-Clo showed no hypersensitivity (Supplementary Figure S7).

**FIGURE 6:**
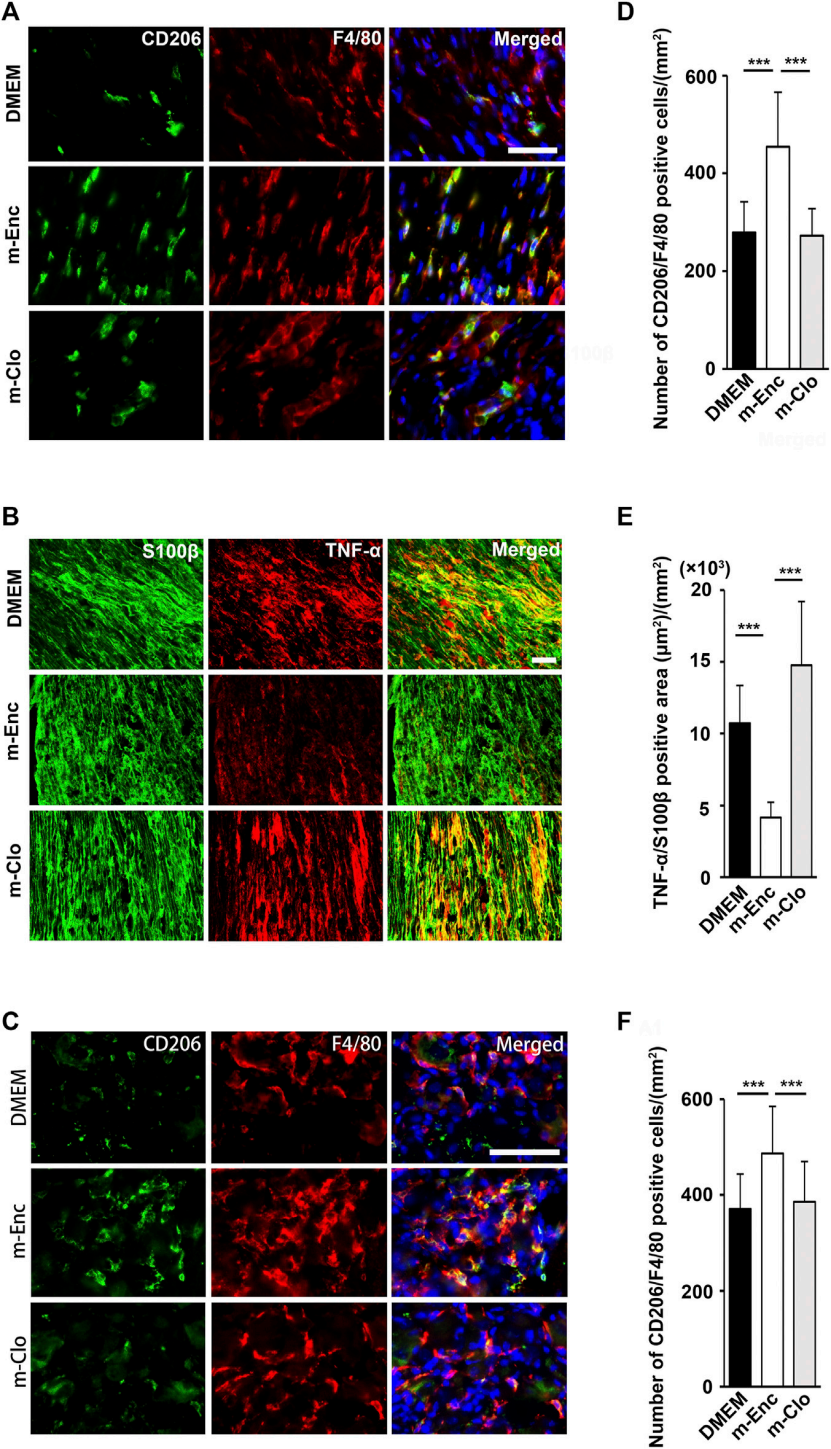
Effects of depletion of SHED-CM-induced M2 on the activation of Schwann cells. **(A)** Representative images of immunofluorescent staining of **(A)** CD206, F4/80 and **(B)** TNF-α, S100β in SCN, and **(C)** CD206, F4/80 in L4/5 DRG 7 days after PSL. Scale bar: 50 μm in **(A,B,C)**. **(D–F)** Quantification analysis of **(D)** CD206^+^ F4/80^+^ cell numbers and **(E)** TNF-α^+^ S100β^+^ area in SCN, and **(F)** CD206^+^ F4/80^+^ cell numbers in L4/5 DRG. Tukey multiple comparisons of means (*n* = 8–10, per group). Data represent the mean ± SD. ***p < 0.001.

**FIGURE 7:**
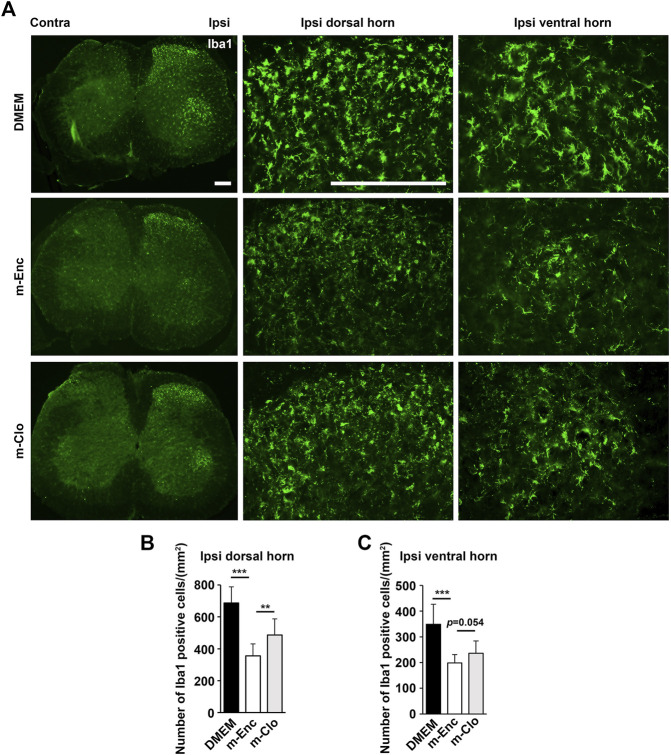
Effects of depletion of SHED-CM-induced M2 on the activation of microglia. **(A)** Representative images of immunofluorescent staining of Iba1 in the ipsilateral dorsal and ventral horn of L3/4 SP 7 days after the PSL. **(B,C)** Quantification analysis of Iba1^+^ cell numbers. Tukey multiple comparisons of means (*n* = 8–10, per group). Data represent the mean ± SD. **p < 0.01, ***p < 0.001.

Furthermore, immunohistochemical analysis showed that m-Clo, but not m-Enc, reduced the number of CD206^+^ F4/80^+^ macrophages in SCN and DRG while increasing TNF-α^+^ S100^+^ proinflammatory Schwann cells in SCN and Iba1^+^ activated microglia in the spinal cord (Figures 6, 7). These results suggest that SHED-CM-induced M2 macrophages are crucial for the suppression of proinflammatory response and antinociceptive activity.

### CM From M2 Induced by SHED-CM Suppresses Proinflammatory Activities of Schwann Cell *In Vitro*

**FIGURE 8:**
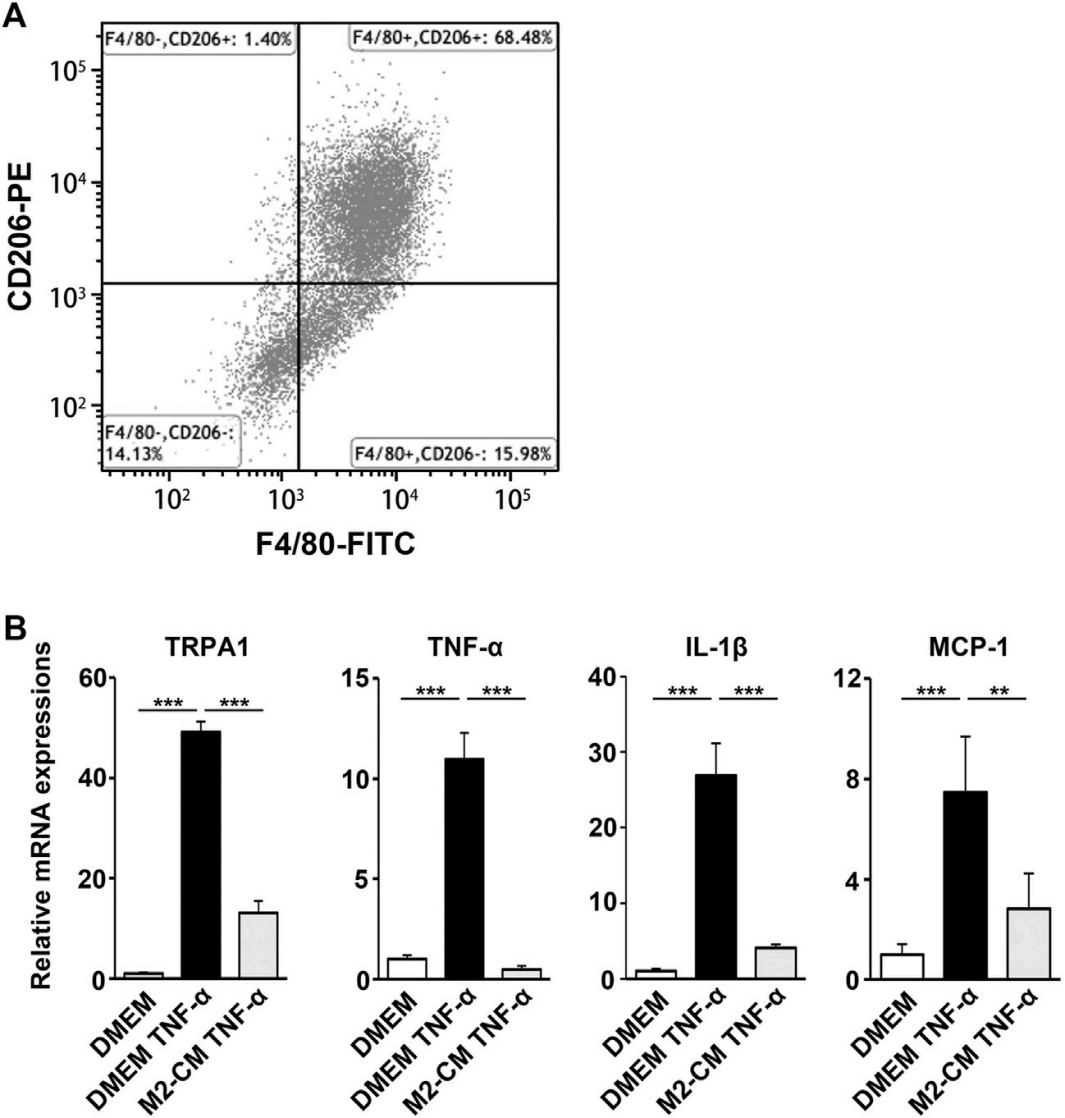
M2-CM suppresses the pro-nociceptive properties of TNF-α-activated Schwann cells *in vitro*. **(A)** Flow cytometry analysis of SHED-CM-induced M2 polarization. Mouse bone marrow-derived macrophages were treated with SHED-CM for 24 h and stained with F4/80 and CD206 antibodies. **(B)** qPCR analysis of human Schwann cells treated with TNF-α with or without M2-CM. Notably, M2-CM strongly inhibited the TNF-α-mediated elevation of pro-nociceptive factors and channels in Schwann cells. Tukey multiple comparisons of means (*n* = 3, per group). Data represent the mean ± SD; **p < 0.01, ***p < 0.001.

We next examined the biological activity of the secretion from M2 induced by SHED-CM. MCSF-treated bone marrow cells differentiated into macrophages, which were subsequently stimulated with SHED-CM for 24 h. In addition, more than 68.48% of the cells differentiated into CD206^+^ F4/80^+^ M2 macrophages following the same procedure (Figure 8A). We harvested the secretion from M2 induced by SHED-CM as M2-CM.

Schwann cells first detect nerve injury and play a critical role in the development and maintenance of NP. Under proinflammatory conditions, they express cytokines TNF-α and IL-1β, chemokine MCP-1, and transient receptor potential ankyrin 1 (TRPA1) channels, which accelerate neuroinflammation and mechanical allodynia. We treated human Schwann cells with TNF-α and M2-CM or DMEM for 24 h and analyzed the gene expression profile. The TNF-α treatment increased the expression of TRPA1, TNF-α, IL-1β, and MCP-1, whereas the M2-CM treatment strongly suppressed this upregulation (Figure 8B).

### M2-CM Attenuates NP *In Vivo*

**FIGURE 9:**
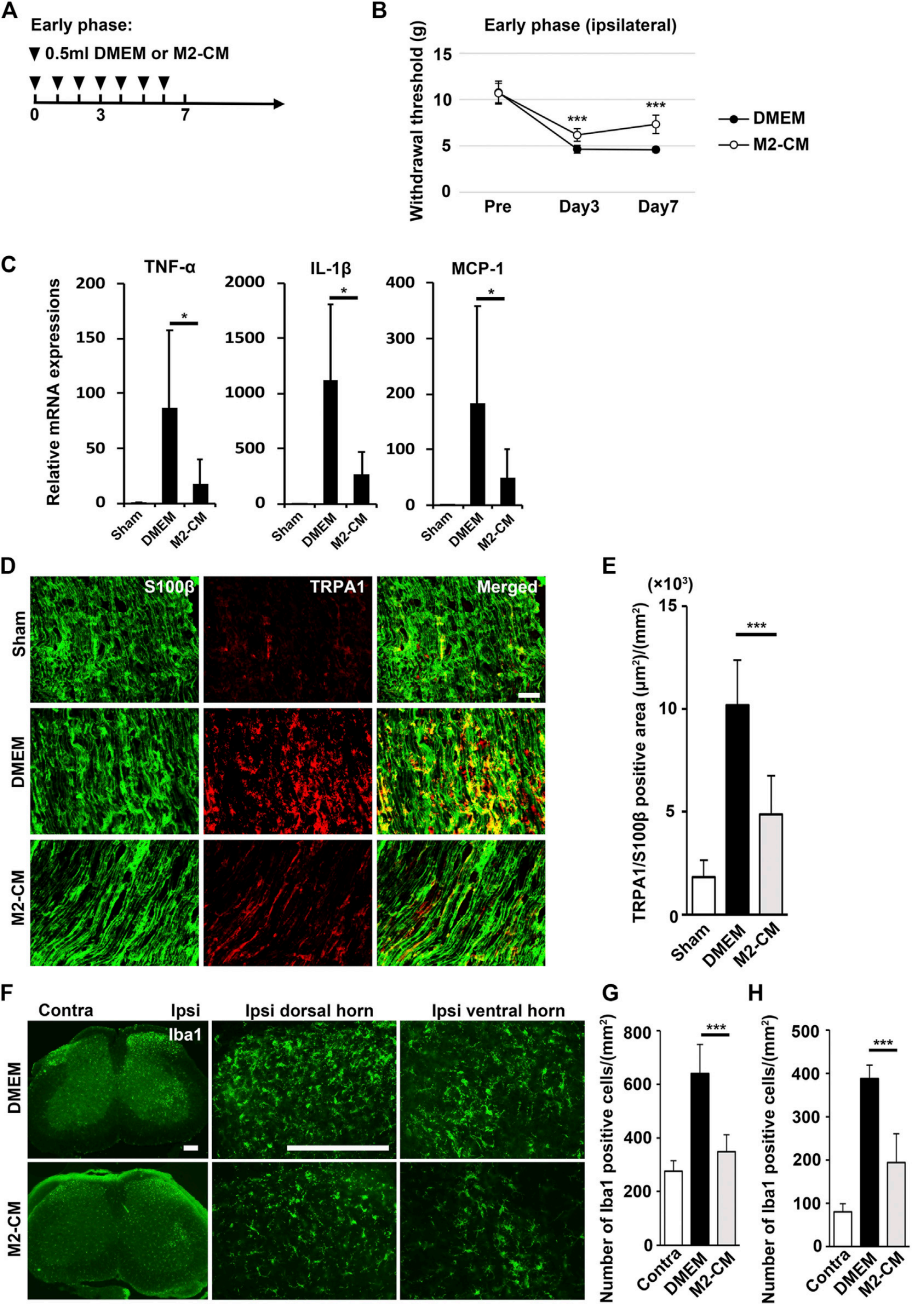
Analgesic effect of M2-CM. **(A)** Time course of M2-CM treatment in the early phase model. **(B)** The ipsilateral paw withdrawal thresholds in the early phase model. Student’s *t*-test (*n* = 10 in each group). **(C)** qPCR analysis of the sciatic nerves 3 days after PSL for the indicated mRNAs. Student’s *t*-test (*n* = 4–6). **(D)** The immunofluorescent staining of TRPA1, S100β in SCN. Scale bar: 50 μm. **(E)** Quantitative analysis of the TRPA^+^ S100β^+^ area. **(F)** The immunofluorescent staining of Iba1 in the ipsilateral horn of the L3/4 SP. Scale bar: 200 μm. **(G,H)** Quantitative analysis of the number of Iba1^+^ cells. Student’s *t*-test (*n* = 7–9, per group). Data represent the mean ± SD; ***p < 0.001.

To examine the antinociceptive activity of M2-CM, we administered it to the PSL mice. We found that M2-CM, but not DMEM, prevented PSL-induced allodynia and proinflammatory responses in the SCN (Figures 9A–C). In addition. M2-CM also suppressed TRPA1 expression in Schwann cells *in vivo* (Figures 9D,E). The results also showed that Iba1^+^ positive cells on the ipsilateral side of L3/4 were extensively decreased in the M2-CM group compared with that in the DMEM group (Figures 9F–H). Collectively, these results suggest that SHED-CM suppressed the neuroinflammation and mechanical allodynia in part through the effect of M2.

### The Possible Therapeutic Factors in SHED-CM Attenuate NP *In Vivo*

**FIGURE 10:**
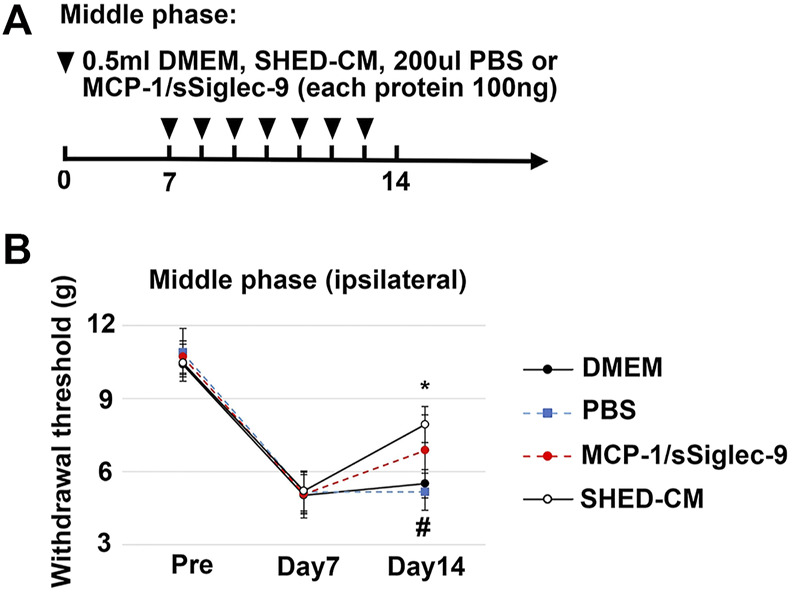
Analgesic effect of MCP-1/sSiglec-9. **(A)** Time course of MCP-1/sSiglec-9 treatment in the middle phase model. **(B)** The ipsilateral paw withdrawal thresholds in the middle phase model. Tukey’s multiple comparisons of means (*n* = 5 in each group). *p < 0.05, for the SHED-CM vs. DMEM. # *p* < 0.05 for the MCP-1/sSiglec-9 vs. PBS.

To confirm the therapeutic effects of a set of M2 inducers, MCP-1 and sSiglec-9, in SHED-CM, were administered to the PSL mice intravenously. We found that, in the middle phase setting, the thresholds (von Frey test) of the right hindpaw of the MCP-1/sSiglec-9 group on day 14 was 7.32 ± 1.75 g, which was significantly higher than that of the DMEM and PBS groups but was lower than that of the SHED-CM group (Figures 10A,B). These results suggest that the promising analgesic ability of SHED-CM might partly rely on MCP-1/sSiglec-9.

## Discussion

This study reports the potential of SHED-CM for the treatment of NP. Intravenous administration of SHED-CM in the early, middle, and late phases of PSL mice prevented nociceptive responses, indicating the potential of SHED-CM to inhibit both development and maintenance of NP. Furthermore, SHED-CM also prevented the deficits of locomotor function caused by PSL. In agreement with the results of the behavior analysis, SHED-CM decreased the number of activated microglia highly expressing Iba1 in both dorsal and ventral hone of spinal cord. Intriguingly the effects of SHED-CM for NP and motor function disappeared 7 and 2 days after the last SHED-CM injection, respectively. The qPCR and immunostaining analysis revealed that SHED-CM treatment induced the anti-inflammatory M2 macrophages in injured SCN and DRG and suppressed the PSL-induced proinflammatory conditions in SCN and glial activation in Schwann cells. Detail mechanisms how SHED-CM induces M2 in DRG and SCN are still elusive. In healthy mice, we found that SHED-CM treatment induced no or little M2 macrophages in DRG and SCN (data not shown). The data suggest that recruitments of pro-inflammatory M1 macrophages to injury tissue is prerequisite for SHED-CM-induced M2 induction. In addition, as shown in Figure 8, SHED-CM was able to induce M2 polarity of bone marrow macrophages *in vitro*. We speculate that primary mechanism of SHED-CM in M2-induction is a promotion of the M2 differentiation of macrophages in DRG and SCN.

Our data showed that m-Clo treatment negated the analgesic effect of SHED-CM. M-Clo seems to be able to deplete mannose receptor (CD206) positive M2 macrophages recruited into the injured SCN, however it has been reported that dendritic cells and nonvascular endothelial cells also express CD206 (Martinez-Pomares, 2012). It is elusive whether CD206-positive cells other than M2 are involved in the analgesic effects of SHED-CM. Importantly we found that the secretome from M2-induced by SHED-CM directly inhibited the proinflammatory/pain-inducing property of Schwann cells and restored NP after PSL. Taken together, our data suggest that the SHED-CM treatment prevents NP mainly through the induction of the analgesic M2.

M2 has been considered to play a crucial role in ameliorating NP (Kiguchi et al., 2017). However, a few studies have investigated the detailed mechanisms by which M2 inhibits pain. It has been reported that perineural injection of IL-4 upregulated the expression of M2 molecules in the SCN in the PSL model, which exhibited an analgesic effect by inhibiting the expression of proinflammatory cytokines and chemokines in the injured SCN (Kiguchi et al., 2015). Moreover, a recent study has shown that IL-4-induced M2 produced opioid peptides, which activated the peripheral opioid receptors and, thereby, ameliorated pain (Celik et al., 2020). These studies showed that IL-4-induced M2 inhibit NP by paracrine mechanisms, suggesting that M2-CM prepared by IL-4 induced M2 also exhibits analgesic activity.

In this study, we demonstrated that the M2-CM treatment directly suppressed the expression of TRPA1 as well as of proinflammatory cytokines and chemokines in TNF-α-activated human Schwann cells and effectively inhibited PSL-induced pain. To our knowledge, this is the first study to demonstrate the analgesic effect of M2-CM. Furthermore, it has recently been reported that TRPA1 in Schwann cells contributes to NP by evoking the NADPH oxidase 1-dependent H_2_O_2_ release, wherein TRPA1 silencing in Schwann cells attenuated nerve injury-induced allodynia and neuroinflammation (De Logu et al., 2017). From the findings of the previous and the present study, it is inferred that, in addition to the previously reported analgesic mechanisms of M2, the direct action of M2 against the proinflammatory/pain-inducing property of Schwann cells might play a significant role in the multifaceted analgesic actions of M2.

Furthermore, this study revealed that in injured SCN treated with SHED-CM, the expression of various neurotrophic factors, including NGF, GDNF and TGF-β, were increased. It has been reported that NGF, GDNF and TGF-β are produced by both macrophages and Schwann cells and are the key growth factors involved in nerve repair, regeneration, and proliferation of Schwann cells (Stewart et al., 1995; Yu et al., 2017; Aarao et al., 2018; Liu et al., 2019; Duarte Azevedo et al., 2020). Although NGF are well-known pain-producing substances, in SHED-CM-treated SCN, these factors did not activate the nociceptive response.

Regulation of microglial inflammatory responses in the CNS is important as a therapeutic strategy for motor function as well as NP (Nakajima et al., 2020). In our experiments, microglia in the dorsal and ventral horn of the spinal cord were activated after PSL, and their activation was both suppressed by SHED-CM treatment. Notably, M2 depletion by m-Clo attenuated the anti-inflammatory effects of SHED-CM on microglia. In addition, intravenously administered SHED-CM did not induce anti-inflammatory M2 microglia in the spinal cord (data not shown). These results suggest that most of the anti-inflammatory effects of intravenously administered SHED-CM on the CNS are indirect.

Our previous studies have identified a set of M2 inducers, sSiglec-9 and MCP-1, by secretome analysis of SHED-CM (Matsubara et al., 2015). These studies have shown that neither MCP-1 nor sSiglec-9 alone but the combination of MCP-1 and sSiglec-9 recapitulated the SHED-CM activity to induce M2-like macrophages. In this study, we found that the antinociceptive activity of the MCP-1/sSiglec-9 treatment was significantly inferior to that of SHED-CM. Even after M2 depletion, the threshold for tactile stimuli of the m-Clo group was significantly better than that of the DMEM group. These data indicate the possibility that SHED-CM could have anti-pain factors other than MCP-1/sSiglec-9. These findings are partially supported by those of a previous study (Ogasawara et al., 2020), which characterized the soluble factors in SHED-CM by performing a secretome analysis and reported that SHED-CM contained 51 array proteins that had 10-fold higher expression levels than those detected in Fibro-CM. Furthermore, our LC/MS studies have shown the potential of several other factors, including the neurotrophic factors, to attenuate NP. For instance, the secreted frizzled-related protein 1 (SFRP1), a Wnt antagonist, has been reported as a therapeutic agent for NP because of its anti-inflammatory activity (Tang et al., 2018). It has also been shown that TGF-β inhibits the expression of proinflammatory cytokines and hence suppresses the activation and proliferation of glial cells in the spinal cord in a mouse nerve injury model (Echeverry et al., 2009; Chen et al., 2013). Notably, TGF-β also suppresses nerve injury-induced spinal cord synaptic plasticity and DRG neuronal hyperexcitability (Chen et al., 2015). Recently, the effectiveness of alpha 2 macroglobulin (A2M), a plasma protein, was investigated in the neurogenic thoracic outlet syndrome and other forms of cervical brachial syndrome. It acts as a molecular trap for inflammatory factors, which counteracts inflammation and hence ameliorates pain (Jordan et al., 2020). In addition, hepatocyte growth factor (HGF) with potential angiogenic and neurotrophic properties has been shown to attenuate NP by suppressing pain-related genes, activating transcription factor 3 (ATF3), α2δ1, and colony stimulating factor 1 in DRG neurons (Nho et al., 2018). In diabetic NP, increasing glucose-6-phosphate dehydrogenase in DRGs has been reported to attenuate hindpaw hypersensitivity because of suppression of toll-like receptor 4 (Sun et al., 2019). Although the concentrations of these factors in SHED-CM might be quite low, we believe that the combinatorial effects of these factors in SHED-CM could provide therapeutic benefits to ameliorate NP. Therefore, the roles of these therapeutic factors in SHED-CM-mediated analgesic effects should be investigated in the future.

## Conclusion

In this study, we demonstrate the potential of SHED-CM for treating NP. Although the treatment of NP remains a clinical challenge, our results suggest that increasing M2 macrophages after the administration of SHED-CM could be a promising method to modify the microenvironment in peripheral nerves and, thereby, cure NP.

## Data Availability

The raw data supporting the conclusions of this article will be made available by the authors, without undue reservation.

## Abbreviations

Arg-1, arginase-1; A2M, alpha 2 macroglobulin; ATF3, activating transcription factor 3; BDNF, brain-derived neurotrophic factor; NP, neuropathic pain; CM, conditioned medium; DMEM, Dulbecco’s modified Eagle’s medium; DPSC, dental pulp stem cell; Fibro-CM, fibroblast-CM; GAPDH, glyceraldehyde-3-phosphate dehydrogenase; GDNF, glial cell derived neurotrophic factor; HGF, hepatocyte growth factor; IL-1β, interleukin-1β; iNOS, inducible nitric oxide synthase; M2-CM, conditioned medium derived from M2 macrophage; MCSF, macrophage colony stimulating factor; m-Enc, mannosylated control liposome; m-Clo, mannosylated-Clodrosome; MCP-1, monocyte chemoattractant protein-1; NGF, nerve growth factor; PBS; phosphate-buffered saline; PSL, partial sciatic nerve ligation; SHED, stem cells from human exfoliated deciduous teeth; SHED-CM, conditioned medium derived from stem cells from human exfoliated deciduous teeth; SCN, sciatic nerve; sSiglec-9, secreted ectodomain of sialic acid-binding Ig-like lectin-9; TNF-α, tumor necrosis factor-α; TGF-β, transforming growth factor-β; TRPA1, transient receptor potential ankyrin 1; SFRP1, secreted frizzled-related protein 1.
