# Supplementary material for: Conditioned Medium From the Stem Cells of Human Exfoliated Deciduous Teeth Ameliorates Neuropathic Pain in a Partial Sciatic Nerve Ligation Model
Source: Front Pharmacol. 2022 Mar 31;13:745020. doi: 10.3389/fphar.2022.745020 (PMC9009354; doi:10.3389/fphar.2022.745020)
Supplement: Supplementary file 1 [file DataSheet7.PDF]

Supplemental figure 7

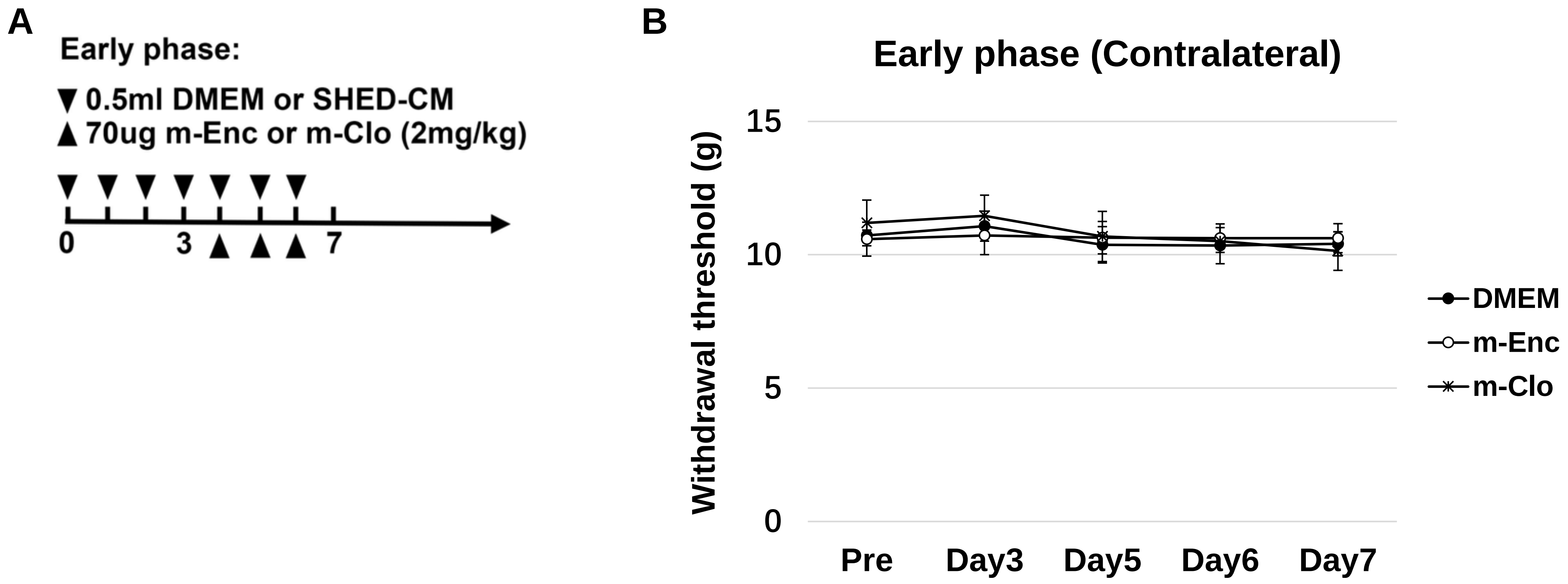

Suppl. Fig 7: Effects of M2 depletion on contralateral side after PSL. (**A**) Time course of M2 depletion in the early phase model. After PSL, mice were daily administered with either 0.5 mL of SHED-CM or DMEM. From day 4 to 6, mannosylated-Clodrosome (m-Clo; for M2 depletion) or mannosylated-Encapsome (m-Enc; negative control) were injected together with SHED-CM. (**B**) The contralateral paw withdrawal thresholds. The paw withdrawal thresholds in the DMEM, m-Enc and m-Clo group were no significant difference (n = 5, per group). Data represent the mean ± SD.
