# Supplementary material for: Conditioned Medium From the Stem Cells of Human Exfoliated Deciduous Teeth Ameliorates Neuropathic Pain in a Partial Sciatic Nerve Ligation Model
Source: Front Pharmacol. 2022 Mar 31;13:745020. doi: 10.3389/fphar.2022.745020 (PMC9009354; doi:10.3389/fphar.2022.745020)
Supplement: Supplementary file 2 [file DataSheet2.PDF]

Supplemental figure 2

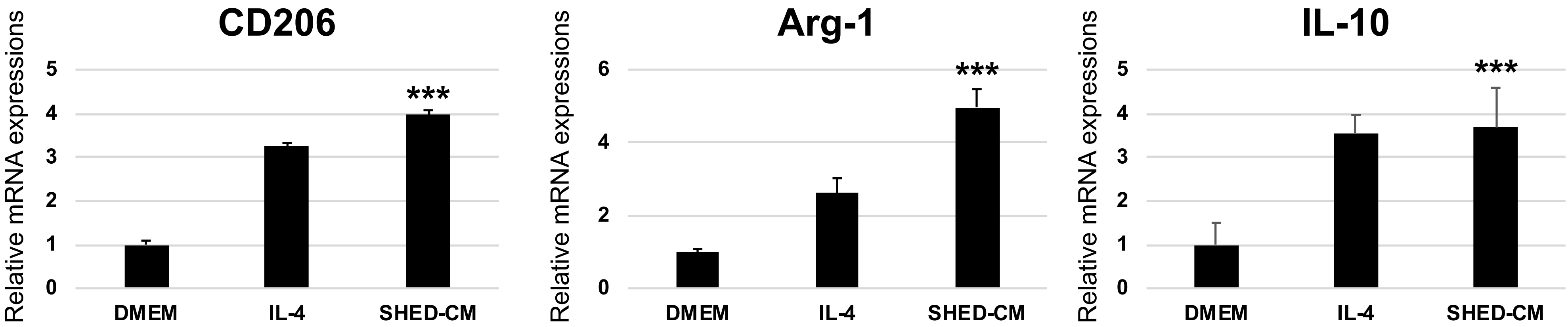

Suppl. Fig 2: Gene analysis of M2-type cells or trophic factors of macrophages incubated with serum-free DMEM, IL-4 or SHED-CM. Results are expressed relative to the levels in the sham-operated model. Student's *t*-test. Data represent the mean ± SD. ***$p < 0.001$, for the DMEM vs. SHED-CM.
