# Supplementary material for: Conditioned Medium From the Stem Cells of Human Exfoliated Deciduous Teeth Ameliorates Neuropathic Pain in a Partial Sciatic Nerve Ligation Model
Source: Front Pharmacol. 2022 Mar 31;13:745020. doi: 10.3389/fphar.2022.745020 (PMC9009354; doi:10.3389/fphar.2022.745020)
Supplement: Supplementary file 3 [file DataSheet4.PDF]

Supplemental figure 4

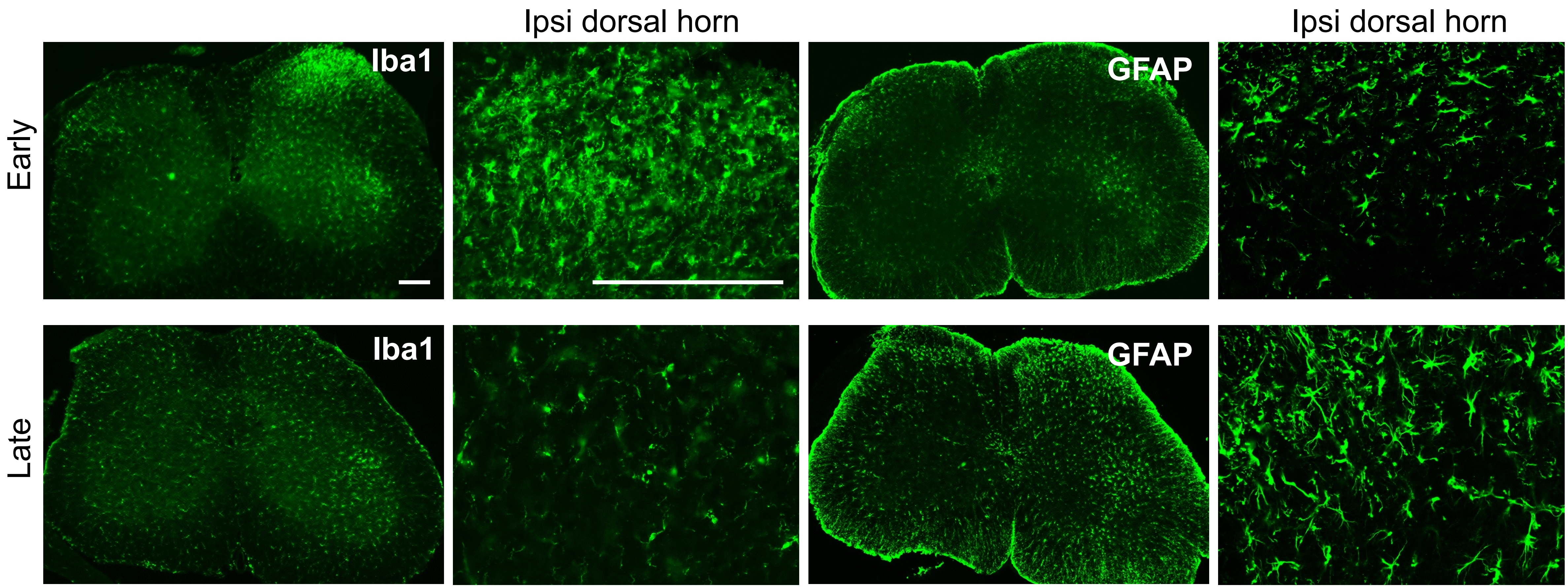

Suppl. Fig 4: Microglia and astrocytes respectively activated in early and late phase. Representative images of immunofluorescent staining of IBA1 and GFAP in spinal cord in spinal cord in early (Day7 after PSL) and late phase (Day21 after PSL) (n=4-5). Scale bar: 200μm.
