# Supplementary material for: Conditioned Medium From the Stem Cells of Human Exfoliated Deciduous Teeth Ameliorates Neuropathic Pain in a Partial Sciatic Nerve Ligation Model
Source: Front Pharmacol. 2022 Mar 31;13:745020. doi: 10.3389/fphar.2022.745020 (PMC9009354; doi:10.3389/fphar.2022.745020)
Supplement: Supplementary file 4 [file DataSheet6.PDF]

Supplemental figure 6

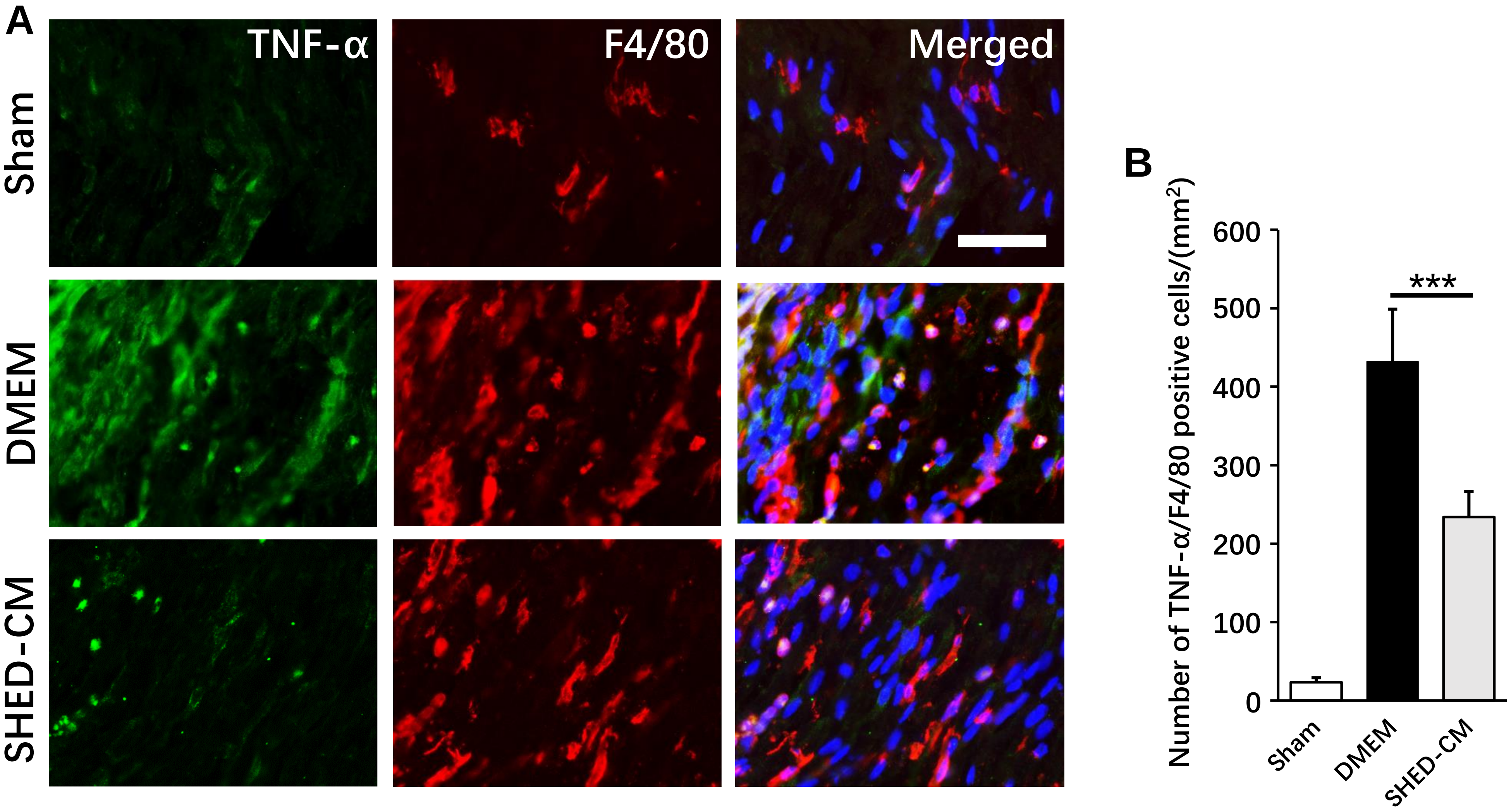

Suppl. Fig 6: SHED-CM treatment reduces M1 macrophages. Images of immunofluorescent staining of TNF-α, F4/80 (**A**) in SCN. Quantification of TNF-α^{+}F4/80^{+} cell numbers (**B**) in SCN. Scale bar: 50 μm. At least 3 non-overlapping sections from 5 animals per group were used to determine the positive cells. Data represent the mean ± SD. Student's *t*-test (n = 5, per group); $^{***}p < 0.001$.
