# Supplementary material for: Conditioned Medium From the Stem Cells of Human Exfoliated Deciduous Teeth Ameliorates Neuropathic Pain in a Partial Sciatic Nerve Ligation Model
Source: Front Pharmacol. 2022 Mar 31;13:745020. doi: 10.3389/fphar.2022.745020 (PMC9009354; doi:10.3389/fphar.2022.745020)
Supplement: Supplementary file 5 [file DataSheet3.PDF]

Supplemental figure 3

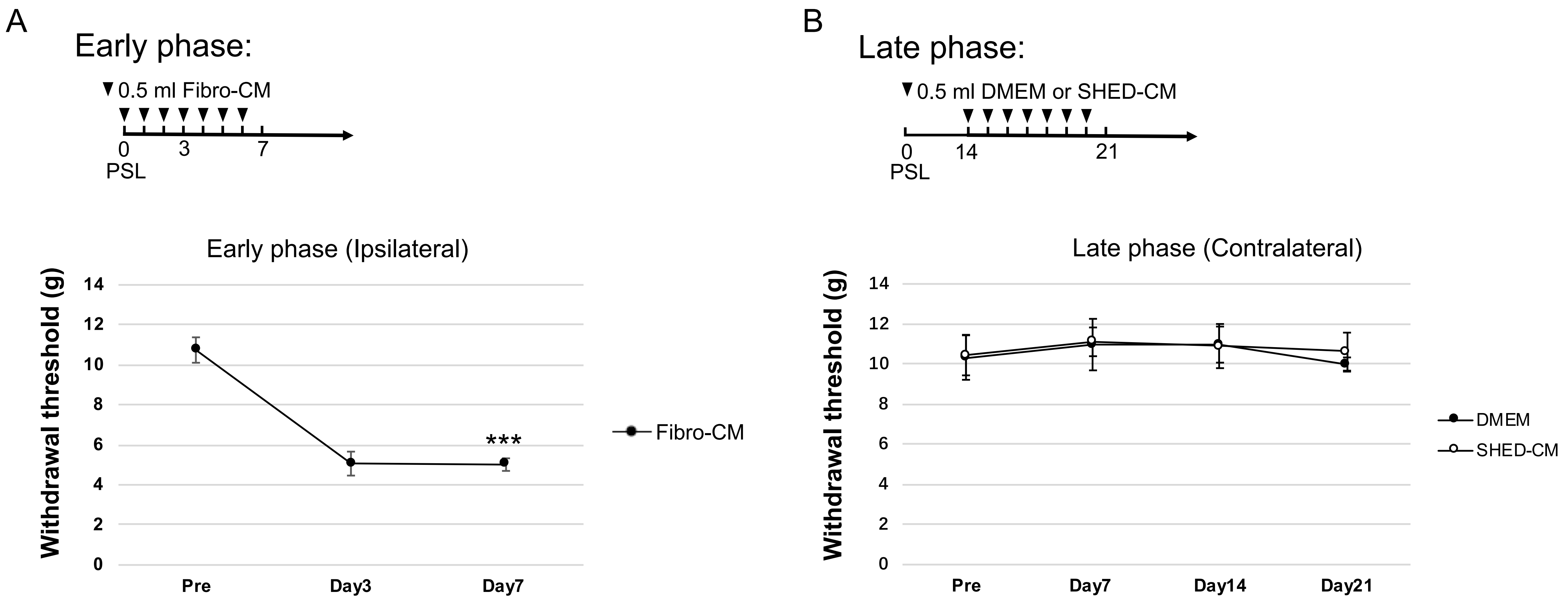

Suppl. Fig 3: Von Frey test of fibroblast-CM treatment in early phase and SHED-CM treatment in late phase. (A) Von Frey test of fibroblast-CM treatment ipsilateral side. Student's *t*-test (n=10). (B) Von Frey test of contralateral side (n=5). Data represent the mean ± SD. ****p* < 0.001, for the Day7 vs. Pre.
