# Supplementary material for: Conditioned Medium From the Stem Cells of Human Exfoliated Deciduous Teeth Ameliorates Neuropathic Pain in a Partial Sciatic Nerve Ligation Model
Source: Front Pharmacol. 2022 Mar 31;13:745020. doi: 10.3389/fphar.2022.745020 (PMC9009354; doi:10.3389/fphar.2022.745020)
Supplement: Supplementary file 6 [file DataSheet5.PDF]

Supplemental figure 5

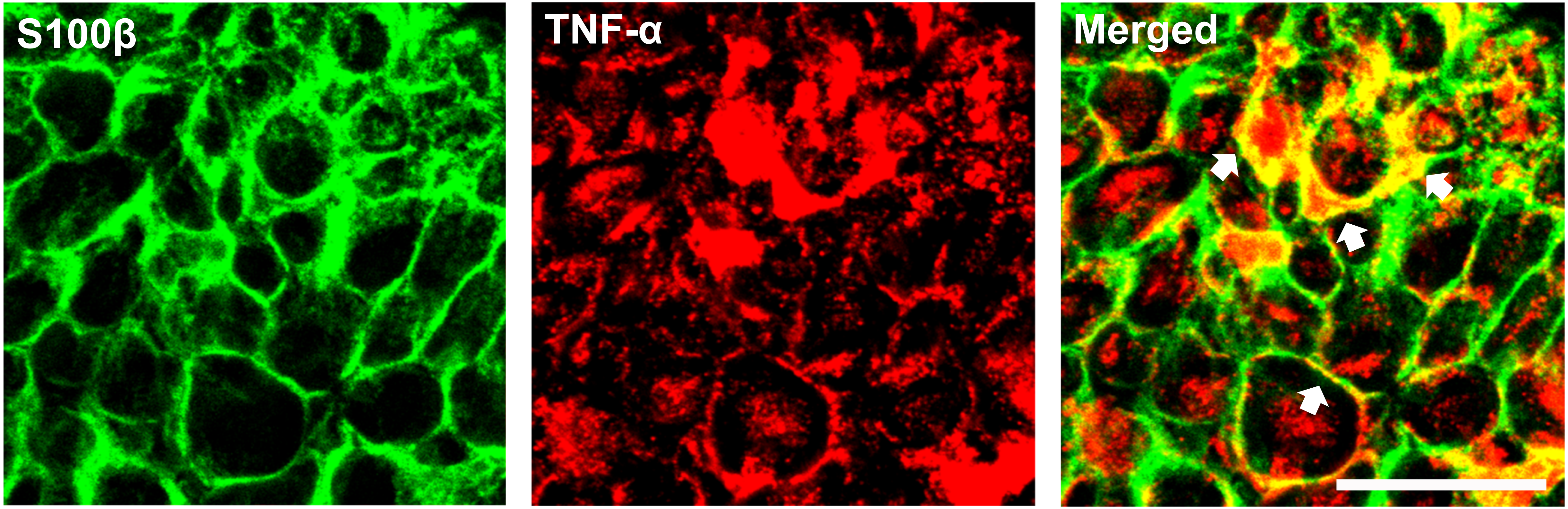

Suppl. Fig 5: Confocal images of immunofluorescent staining of TNF-α, S100β in SCN transverse sections 24h after PSL. Note that S100β signal from Schwann cells overlapped with TNF-α (arrow). Scale bar: 20μm.
