# Supplementary material for: Conditioned Medium From the Stem Cells of Human Exfoliated Deciduous Teeth Ameliorates Neuropathic Pain in a Partial Sciatic Nerve Ligation Model
Source: Front Pharmacol. 2022 Mar 31;13:745020. doi: 10.3389/fphar.2022.745020 (PMC9009354; doi:10.3389/fphar.2022.745020)

Supplemental Figure 1

1. DMEM, SHED-CM and Fibro-CM injection Experimental Design in early phase model.


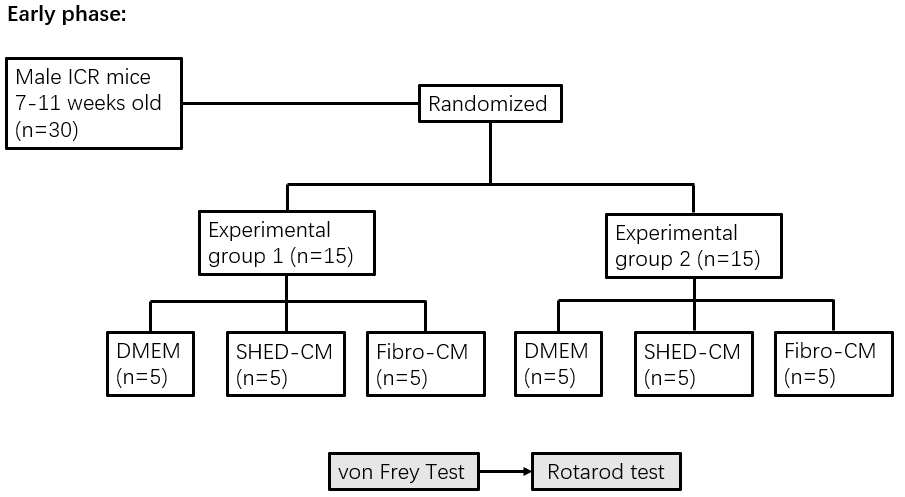


1. DMEM, SHED-CM and Fibro-CM injection Experimental Design in early phase model.


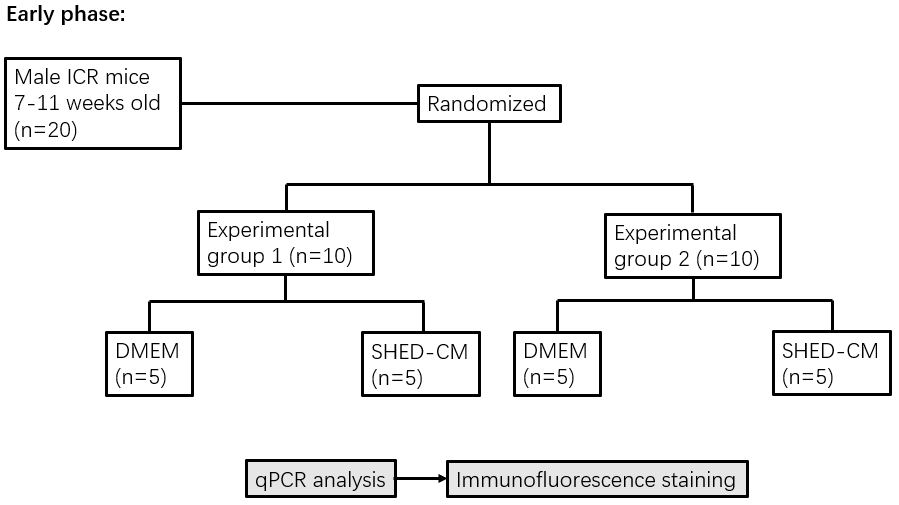


1. DMEM and SHED-CM injection Experimental Design in middle/late phase model.


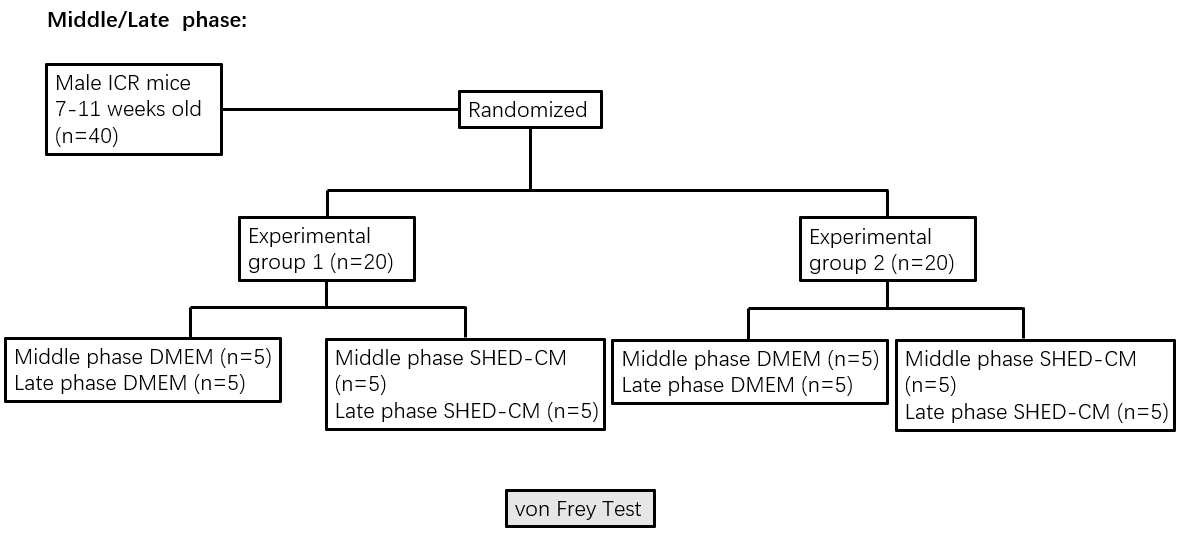


1. M2 macrophage depletion Experimental Design in early phase model.


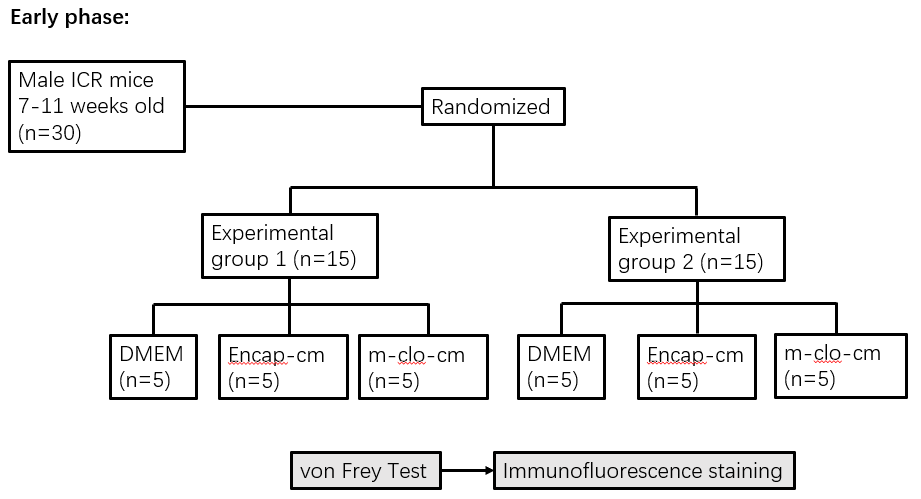


1. DMEM and M2-CM injection Experimental Design in early phase model.


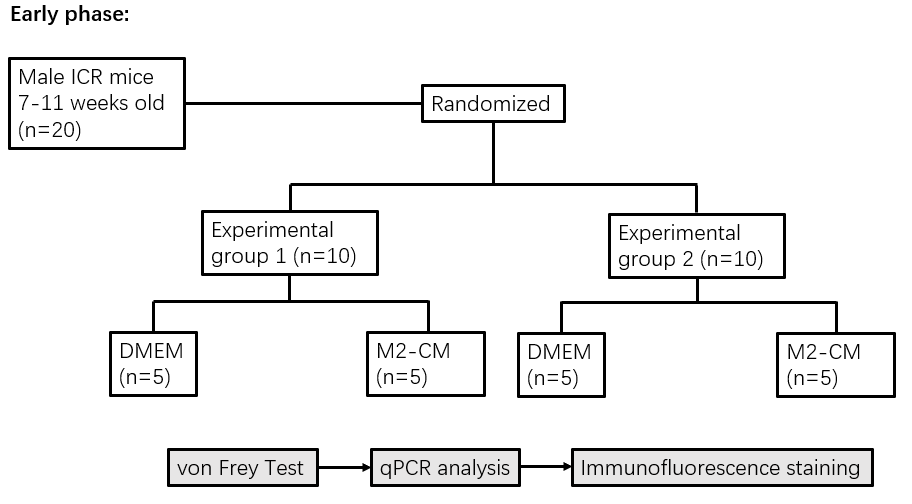


1. DMEM, SHED-CM, PBS and MCP-1/sSiglec-9 injection Experimental Design in middle phase model.


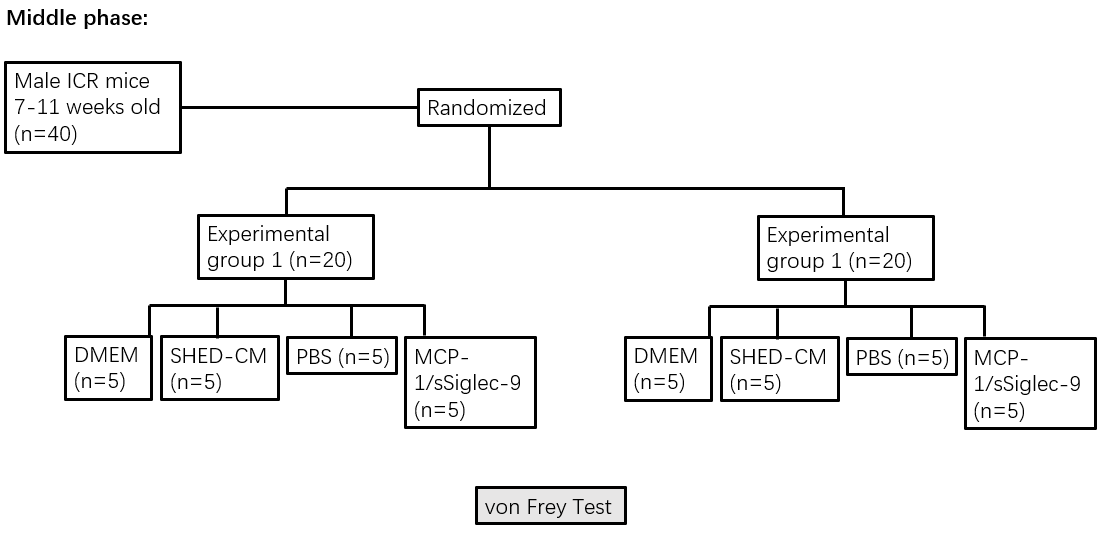

Supplement: Supplementary file 7 [file DataSheet1.DOCX]
